# Asxl1 exerts an antiproliferative effect on mouse lung maturation via epigenetic repression of the E2f1-Nmyc axis

**DOI:** 10.1038/s41419-018-1171-z

**Published:** 2018-11-02

**Authors:** Seungtae Moon, Sun-Kyoung Im, Nackhyoung Kim, Hyesook Youn, Ui-Hyun Park, Joo-Yeon Kim, A.-Reum Kim, So-Jung An, Ji-Hoon Kim, Woong Sun, Jin-Taek Hwang, Eun-Joo Kim, Soo-Jong Um

**Affiliations:** 1 Department of Integrative Bioscience and Biotechnology, Sejong University, Seoul, 05006 Korea; 2 Severance Biomedical Science Institute, Yonsei University College of Medicine, Seoul, 06273 Korea; 3 Department of Anatomy, Korea University College of Medicine, Seoul, 02841 Korea; 4 School of Biological Science, College of Natural Sciences, Seoul National University, Seoul, 08826 Korea; 5 Korea Food Research Institute, Jeonju, Jeonbuk 55365 Korea; 6 Department of Molecular Biology, Dankook University, Chungnam, 31116 Korea

## Abstract

Although additional sex combs-like 1 (ASXL1) has been extensively described in hematologic malignancies, little is known about the molecular role of ASXL1 in organ development. Here, we show that *Asxl1* ablation in mice results in postnatal lethality due to cyanosis, a respiratory failure. This lung defect is likely caused by higher proliferative potential and reduced expression of surfactant proteins, leading to reduced air space and defective lung maturation. By microarray analysis, we identified E2F1-responsive genes, including *Nmyc*, as targets repressed by Asxl1. *Nmyc* and *Asxl1* are reciprocally expressed during the fetal development of normal mouse lungs, whereas *Nmyc* downregulation is impaired in *Asxl1*-deficient lungs. Together with E2F1 and ASXL1, host cell factor 1 (HCF-1), purified as an Asxl1-bound protein, is recruited to the E2F1-binding site of the *Nmyc* promoter. The interaction occurs between the C-terminal region of Asxl1 and the N-terminal Kelch domain of HCF-1. Trimethylation (me3) of histone H3 lysine 27 (H3K27) is enriched in the *Nmyc* promoter upon *Asxl1* overexpression, whereas it is downregulated in *Asxl1*-deleted lung and -depleted A549 cells, similar to H3K9me3, another repressive histone marker. Overall, these findings suggest that Asxl1 modulates proliferation of lung epithelial cells via the epigenetic repression of Nmyc expression, deficiency of which may cause hyperplasia, leading to dyspnea.

## Introduction

The additional sex combs-like (*ASXL*) family is the mammalian homolog of the additional sex combs (*Asx*) gene, which is an enhancer of trithorax group (TrxG) and polycomb group (PcG) proteins in *Drosophila*^1^. There are three members of the *ASXL* family—*ASXL1*, *ASXL2*, and *ASXL3*^2^. ASXL family proteins are chromatin factors that exert diverse effects, including on tumor suppression and development, by modulating gene expression. We previously demonstrated that ASXL1 is primarily located in the nucleus and regulates nuclear hormone receptors in cooperation with either SRC1 as a coactivator or HP1α as a corepressor^3,4^. *Drosophila* Asx complexes with the histone deubiquitinase Calypso (mammalian BAP1) forms a polycomb repressive deubiquitinase (PR-DUB) complex and stimulates removal by Calypso of monoubiquitin from H2A lysine 119 for transcriptional repression^5^. The enzymatic activity of the PR-DUB complex was also examined in mammals using the ASXL family^6^. Additionally, ASXL1 interacts with EZH2, a member of polycomb repressive complex 2 (PRC2) and responds to PRC2-mediated gene silencing^7,8^.

*ASXL1* is frequently mutated in hematological or myeloid malignancies; such mutations are associated with adverse outcomes^9–11^. In contrast, *ASXL1* mutations in solid tumors are rarely reported, and their effects are unclear^2^. Hematopoietic-specific deletion of *Asxl1* or overexpression of an *ASXL1* mutant resulted in myelodysplasia-like syndromes in mice^12–15^. Despite reports of somatic *ASXL1* mutations in leukemia, the mechanism by which ASXL1 exerts effects against cancer is unknown. *Asxl1* ablation leads to significant reduction of H3K27 trimethylation, likely due to impaired interaction with Ezh2, a histone methyltransferase^12^. Although *Bap1* loss also promotes myeloid transformation in mice^16^, the underlying mechanism appears to be different from that of *Asxl1* loss in terms of PRC2 regulation^17^. Germline *ASXL1* mutations are reported in around 50% of patients with Bohring-Opitz syndrome^18,19^. Recent studies using *Asxl1*-null mouse models indicated a critical role for *Asxl1* in development. Depending on the model, *Asxl1* loss causes embryonic lethality and developmental abnormalities, including dwarfism, anophthalmia, microcephaly, kidney podocyte defects, and craniofacial defects^12,14,20,21^. Other investigation using *Asxl1*-null mice reported the potential role of Asxl1 in lung maturation^22^. However, the molecular mechanism by which *Asxl1* loss causes these defects remains unexplored. Recently, we showed that Asxl1 interacts with Akt in mouse embryonic fibroblasts (MEFs) to promote cell proliferation, and *Asxl1* disruption results in cellular senescence by increasing *p16Ink4a* expression *via* Ezh2 inactivation^8^. Intriguingly, this role of Asxl1 in MEF proliferation is the opposite of its function as a tumor suppressor^23^. Therefore, the mechanism underlying this difference in various tissues and at different developmental stages needs to be investigated.

Lung development in mice is divided into five distinct stages according to gestational age: embryonic (E9–11.5), pseudoglandular (E11.5–16.5), canalicular (E16.5–17.5), saccular (E17.5–PN5), and alveolar (PN5–28) stages^24^. During lung maturation, the respiratory air spaces are formed by progressive branching of epithelial-lined airways into the lung mesenchyme^25^. Epithelial cells lining the pulmonary air spaces begin to differentiate into functional Type I and Type II pneumocytes^26^. Type I pneumocytes are squamous and thin, cover about 95% of epithelial air surface, and participate in gas exchange, whereas Type II pneumocytes are granular and cuboidal, and secrete surfactants accompanying with depletion of glycogen content. Genetic and epigenetic defects in these pneumocytes may cause lung diseases including respiratory distress syndrome (RDS) and cancer. Our previous observations that most newborn *Asxl1*-null mice died just after birth prompted us to investigate the underlying mechanism, focusing on the role of Asxl1 in lung development associated with epithelial cell proliferation and differentiation.

Our studies indicated that the dyspnea in *Asxl1*^−/−^ mice is caused by abnormally increased proliferation and reduced maturation of Type ll pneumocytes. The underlying mechanism was identified by microarray analysis, ASXL1-bound complex purification, transcription assay, and chromatin immunoprecipitation. Overall, our data suggest that Asxl1 forms a complex with HCF-1 to repress E2F1-driven *Nmyc* transcription by regulating trimethylation of histone H3 lysine 27 and thus controls the Nmyc-associated proliferation of lung epithelial cells.

## Results

### *Asxl1*-knockout mice show perinatal lethality due to respiratory failure

**Fig. 1:**
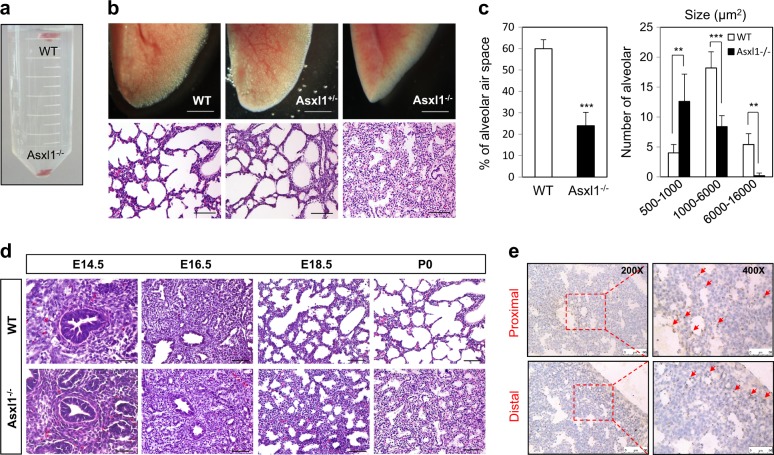
Phenotypic comparison of WT and *Asxl1*^−/−^ littermates. **a** Phenotype of a postnatal day 0 (P0) lung after birth. The P0 lung was examined while it was floating in PBS. **b** The morphology of lung tissue and alveoli in WT and Asxl1^−/−^ at P0. Whole lungs (scale bar, 50 μm) and H&E-stained lungs (scale bar, 100 μm) are shown. **c** Percentages of alveolar air space and alveolar size in WT and *Asxl1*^−/−^ mice. Images were quantitated by Leica Application Suite (LAS) software (Leica, Wetzlar, Germany). Data are means ± SD (*n* = 5, ^**^P < 0.01, ^***^P < 0.001). **d** H&E staining of lung sections from E14.5, E16.5, E18.5, and P0 (scale bar, 100 μm). **e** Distribution of *Asxl1* mRNA in lung epithelium and mesenchyme. In situ hybridization using RNAscope was performed on a sagittal section of an E18.5 lung

To determine the physiological function of Asxl1, we generated *Asxl1* homozygous mice (*Asxl1*^−/−^) (Supplementary Figure S1a)^21^. Inactivation of the *Asxl1* gene was confirmed by Western blotting (WB) (Supplementary Figure S1b) and reverse transcription-quantitative polymerase chain reaction (RT-qPCR) (Supplementary Figure S1c). Before birth, embryos from *Asxl1*^+/−^ intercrosses showed the expected Mendelian ratio (Supplementary Table S1). However, *Asxl1*^−/−^ mice died within 1.5 h of birth due to respiratory failure (Supplementary Figure S1d). In addition to cyanosis, *Asxl1*^−/−^ neonates showed a small body and lung size, anophthalmia, microcephaly, and cleft palates compared to wild-type (WT) neonates after birth (Supplementary Figure S1a, e, and f). Considering the intact structure of the trachea and heart in mutant mice (Supplementary Figure S1g–i), we hypothesized that the respiratory failure arose from defects in lung development. The lung of a WT neonate was well-inflated and floated in phosphate-buffered saline (PBS), whereas the *Asxl1*^−/−^ lung sank (Fig. 1a). As shown by hematoxylin and eosin (H&E) staining, the homozygous *Asxl1*^−/^^−^ lung failed to inflate with air because of its thicker alveolar wall (Fig. 1b), smaller air space, and more numerous small alveoli (Fig. 1c) compared to the WT or the heterozygote. Anatomical changes in alveoli were analyzed from E14.5 to P0 during embryonic lung development. Until E16.5, no significant difference between the WT and *Asxl1*^−/−^ mice was observed. However, alveoli of the *Asxl1*^−/−^ lung at E18.5 and P0 showed a remarkably reduced aerated space, which was compacted with immature-looking cuboidal cells (Fig. 1d). In situ hybridization (ISH) using an E18.5 embryo indicated that *Asxl1* was expressed throughout the lung epithelium and mesenchyme region (Fig. 1e and Supplementary Figure S1j). Insertion of the *LacZ* cassette in the *Asxl1* allele allows to monitor the expression of *Asxl1* gene by X-gal staining^21^. Mutant lungs at various embryonic stages were stained at both epithelium and mesenchyme regions, suggesting ubiquitous expression of *Asxl1* during lung development (Supplementary Figure S1k). This *Asxl1* expression pattern was further confirmed using isolated epithelial and mesenchymal cells at E18.5 (Supplementary Figure S1l), and their markers *Ccsp* and *Vimentin* (Supplementary Figure S1m). Overall, these data suggest that Asxl1, expressed in lung, is critical for proper alveolar formation in the saccular phase of lung development.

### *Asxl1* loss increases the proliferation of lung epithelial cells

**Fig. 2:**
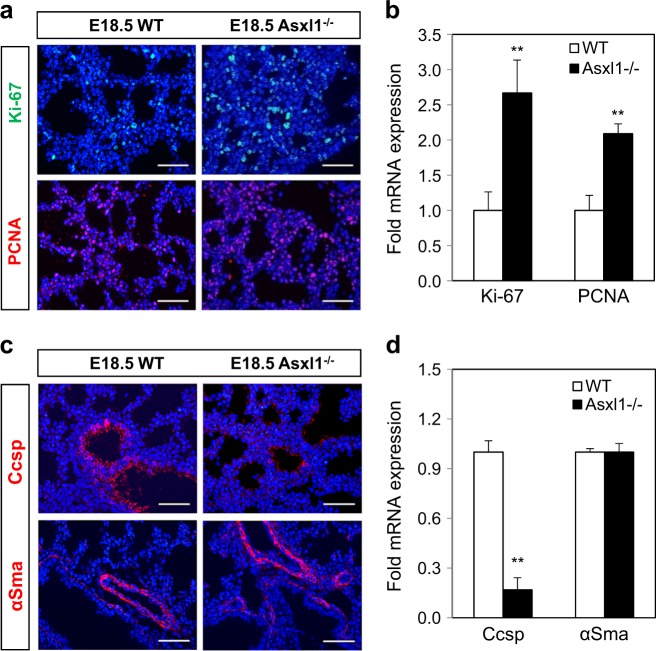
Effect of Asxl1 on the proliferation of lung epithelial cells. **a** Cell proliferation was determined by immunostaining with Ki-67 and PCNA at E18.5. Scale bar, 100 μm. **b** mRNA expression levels were measured by RT-qPCR. **c** Differentiation was assayed using antibodies against the lung epithelial marker Ccsp and the muscle marker αSma at E18.5. **d** mRNA expression levels are shown. Data are means ± SD (*n* = 6, ^**^P < 0.01)

Abnormality of lung alveolar structure might be related to altered cell proliferation, apoptosis, or differentiation. Cell proliferation was measured by staining for the two proliferation markers Ki-67 and proliferating cell nuclear antigen (PCNA). The protein and mRNA levels of Ki-67 and PCNA in *Asxl1*-deleted lungs were significantly higher than those in WT lungs (Fig. 2a, b), but there was no difference between the distal and proximal regions (Supplementary Figure S2a, b). No significant change in apoptosis was detected by terminal deoxynucleotidyl transferase dUTP Nick-End Labeling (TUNEL) assay (Supplementary Figure S2c, d), suggesting that the thickened alveolar wall results from the increased proliferation. Further co-immunostaining with antibodies against PCNA and a lung epithelial marker Nkx2.1 indicated that higher proliferation rate in *Asxl1*-null lung is likely due to the increased proliferation of epithelial cells (Supplementary Figure S2e). To examine the role of *Asxl1* ablation in the differentiation of airway cells, the expression of Club cell secretory protein (Ccsp, also known as Cc10, an airway epithelial marker) and alpha smooth muscle actin (αSma) was monitored. As shown in Fig. 2c, the numbers of Ccsp-positive airway epithelial cells were dramatically decreased in E18.5 *Asxl1*^−/−^ lung, whereas no significant change in those positive for the muscle marker αSma was observed. The mRNA level of *Ccsp* was also downregulated at E18.5 (Fig. 2d) and P0 (Supplementary Figure S2f). In addition, we observed the upregulation of Sox9, a marker of distal epithelial progenitor cells, in *Asxl1*-null E18.5 lung by immunostaining (Supplementary Figure S2g) and RT-qPCR (Supplementary Figure S2h). It has been reported that Sox9 downregulation starting at E16.5 is concurrent with terminal differentiation of Type I and II alveolar cells^27^. Further ex vivo organ culture using E10.5 and E12.5 lungs indicated no effect of *Asxl1* ablation on branching morphogenesis (Supplementary Figure S2i, j). Overall, these data suggest that the defective phenotype of *Asxl1*-null lungs is due to the abnormally increased proliferation of epithelial cells, associating with dysregulated expression of Ccsp and Sox9.

### Asxl1 is critical for lung alveolar maturation

**Fig. 3:**
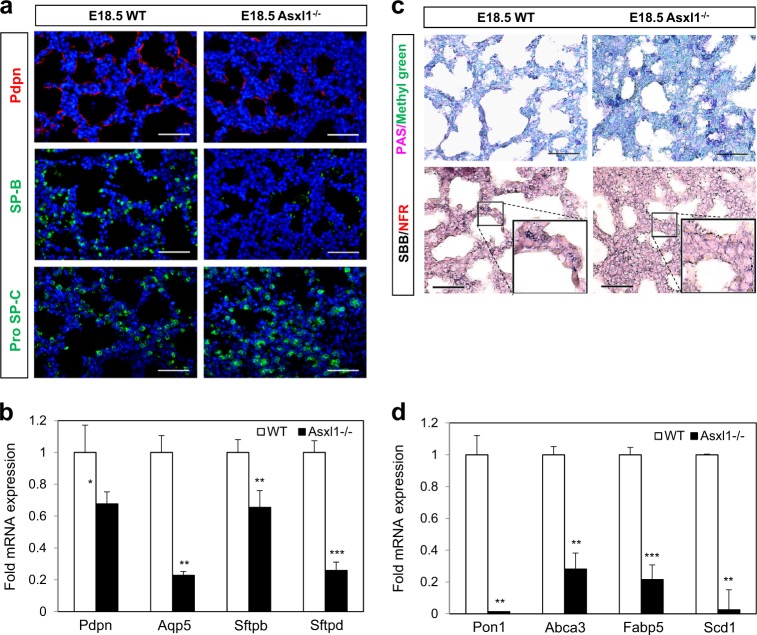
Effects of Asxl1 on the maturation of alveolar Type l and Type ll epithelial cells. **a** Immunostaining for mature Type l (Pdpn), Type ll (SP-B), and immature Type ll (proSP-C) markers at E18.5. Scale bar, 100 μm. **b** mRNA levels of Type l and Type ll cell markers at E18.5 determined by RT-qPCR. Data are means ± SD (*n* = 3, ^*^P < 0.05, ^**^P < 0.01, ^***^P < 0.001). **c** Periodic acid–Schiff (PAS) and Sudan Black B (SBB) staining. Scale bar, 100 μm. **d** Quantification of phospholipid homeostasis markers by RT-qPCR. Data are means ± SD (*n* = 3, ^**^P < 0.01, ^***^P < 0.01)

The dysregulated expression of Ccsp and Sox9 in *Asxl1*-null lungs prompted us to investigate the role of Asxl1 in lung maturation using other epithelial differentiation markers. The WT lung at E18.5 showed well-differentiated Type I and II pneumocytes expressing T1α (*Pdpn*) and surfactant protein-B (SP-B, coded from *Sftpb* gene), whereas the *Asxl1*^−/−^ lung was compacted with precursor cells lacking Pdpn and SP-B expression, but heavily expressing proSP-C (Fig. 3a). Subsequent RT-qPCR analysis revealed a significant reduction in the levels of *Pdpn*, *Aqp5*, *Sftpb*, and *Sftpd* (Fig. 3b). During the morphological and functional maturation of Type II cells, the reserved glycogen is converted into phospholipids to produce lamellar bodies, which are composed of surfactant lipids and proteins^28^. The increased proSP-C staining in *Asxl1*^−/^^−^ lungs indicated immature Type II pneumocytes (Fig. 3a). To confirm this, we performed periodic acid–Schiff (PAS) staining, which enables visualization of glycogen-rich cells. Indeed, the number of PAS-positive cells in the alveolar epithelium of the *Asxl1*^*−*^^/−^ lung was greater than that in the WT lung (Fig. 3c). Additionally, the percentage of glycogen-rich cells in the proximal and distal regions of the *Asxl1*^−/−^ lung was twofold higher than that in the WT control lung (Supplementary Figure S3a, b). Further, phospholipid deposits, which are indicative of Type II cell maturation, were assayed by Sudan Black B (SBB) staining (Fig. 3c). Consistent with the higher glycogen staining in *Asxl1*^−/^^−^ lung, less phospholipid staining was observed, suggesting that Type II pneumocytes in *Asxl1*^−/−^ alveoli are defective in maturation. Finally, this abnormal maturation was confirmed by measuring the expression of genes involved in lung lipid homeostasis. These genes were significantly downregulated in *Asxl1*^−/−^ lungs (Fig. 3d). Overall, we conclude that Asxl1 plays an essential role in alveolar maturation during fetal lung development.

### E2F1-responsive genes, including *Nmyc*, are targets of Asxl1

**Fig. 4:**
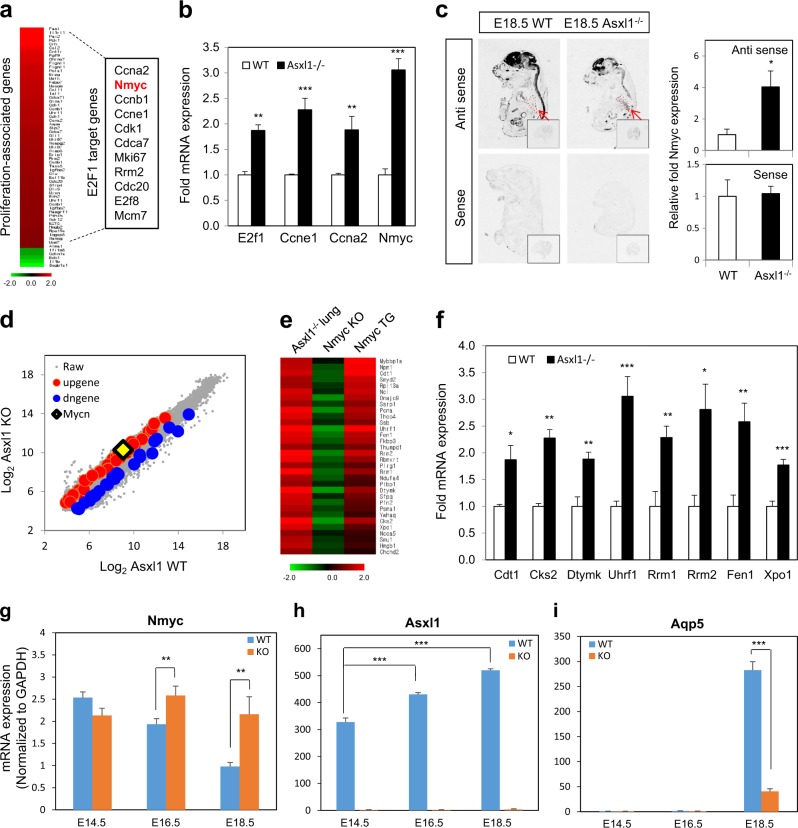
Genome-wide analysis of genes influenced by *Asxl1* deletion. For mRNA quantification, three RT-qPCRs were independently performed. Data are means ± SD (^*^P < 0.05, ^**^P < 0.01, ^***^P < 0.001). **a** Clustering analysis of genes associated with cell proliferation, including E2F target genes. Genes altered more than twofold were clustered. **b** Validation of mRNA levels. Four genes were selected and subjected to RT-qPCR. **c** mRNA level of *Nmyc*, visualized by radio ISH in an E18.5 mouse. Sagittal section of whole body and horizontal section of isolated lung at E18.5. Densities were quantitated. Data are means ± SD (*n* = 3, ^*^P < 0.05). **d** Expression pattern of Nmyc in WT and *Asxl1*^−/−^ lungs. **e** Clustering analysis of genes altered in the *Asxl1*^−/−^ lung and *Nmyc* TG and KO lungs. **f** Validation of the mRNA levels of Nmyc-target genes. **g** Effect of Asxl1 on the mRNA level of Nmyc during fetal lung development. **h** Expression of Asxl1 during lung development. **i** Effect of Asxl1 on the mRNA level of *Aqp5*, a marker of Type I cells

To determine whether *Asxl1* loss alters lung gene expression profiles, we performed a microarray analysis of WT and *Asxl1*^−/^^−^ E18.5 lungs. Subsequent analysis of the expression profiles identified 1307 genes, comprising 1289 upregulated and 18 downregulated genes, with a greater than twofold change in expression in *Asxl1*-null lungs, strongly suggesting a role for Asxl1 in gene repression. Among these genes, the expression levels of cell cycle-, cell proliferation-, and apoptosis-associated genes were significantly altered (Supplementary Table S2 and Supplementary Figure S4a). Clustering analysis of cell proliferation-related genes showed that E2f1 target genes are highly upregulated in *Asxl1*^−/−^ lungs (2.0 extension *Plu*0.05) (Fig. 4a). Further gene set enrichment analysis (GSEA) supported a repressive role for Asxl1 in the expression of genes associated with cell proliferation and E2f1 target genes (Supplementary Figure S4b, c). Upregulation of the mRNA levels of various E2f1 target genes, including *Nmyc*, in E18.5 *Asxl1*^−/−^ lungs, was confirmed by RT-qPCR (Fig. 4b and Supplementary Figure S4d). Additionally, upregulation of the *Nmyc* mRNA level in *Asxl1*-deleted lungs was demonstrated by ISH (Fig. 4c). These observations were reproduced in *ASXL1*-depleted A549, adenocarcinomic human alveolar basal epithelial cells, using adenoviral expression of shASXL1 (Supplementary Figure S4e). However, *Asxl1* overexpression by adenoviral infection of Flag-Asxl1 in A549 cells resulted in downregulation of some E2F1 target genes, including *Nmyc*, but it did not affect the expression of *CDC25A* and *CDC6* (Supplementary Figure S4f). Microarray analysis identified *Nmyc* as one of the upregulated genes associated with cell cycle progression upon *Asxl1* disruption (Fig. 4d). *Nmyc* is also a target of E2F1 in neuroblastomas^29^. *Nmyc* overexpression or conditional deletion is driven by the surfactant *SP-C* promoter/enhancer in mouse lung epithelium, and subsequent genome-wide approaches have provided information on the role of Nmyc in lung development at the transcriptional level^30^. A tight correlation between Nmyc and Asxl1 is suggested by the upregulation of *Nmyc* in *Asxl1*^-/-^ lungs, the similarities in the phenotypes of *Nmyc*-overexpressing and *Asxl1*-deleted lungs, and the GSEA of Nmyc-target genes (Supplementary Figure S4g). To support this observation, we compared our microarray data with those of previous studies using *Nmyc* transgenic (TG) and knockout (KO) mice^30,31^. The expression pattern of genes upregulated in *Asxl1*^−/−^ lungs was opposite to that of genes regulated by *Nmyc* KO but similar to genes regulated by *Nmyc* overexpression (Fig. 4e and Supplementary Table S3). Significant upregulation of Nmyc-target genes in *Asxl1*^−/−^ lungs was determined by RT-qPCR (Fig. 4f). As reported^30^, *Nmyc* expression declines from E14.5 to E18.5 during normal lung development, whereas *Nmyc* expression was elevated in *Asxl1*-deleted lungs (Fig. 4g). In contrast to *Nmyc*, *Asxl1* expression increases from E14.5 to E18.5 (Fig. 4h). Downregulation of *Nmyc* is accompanied by upregulation of markers of differentiated epithelial cells (*Aqp5, Sftpa, Sftpb*, and *Sftpc*), which were downregulated in *Asxl1*-deleted lungs compared to WT lungs (Fig. 4i and Supplementary Figure S4h). Taken together, these data suggest that the expression of *Nmyc* is repressed by Asxl1 in the fetal lung at late stages of development.

### ASXL1 represses Nmyc expression via the E2F1-binding site

**Fig. 5:**
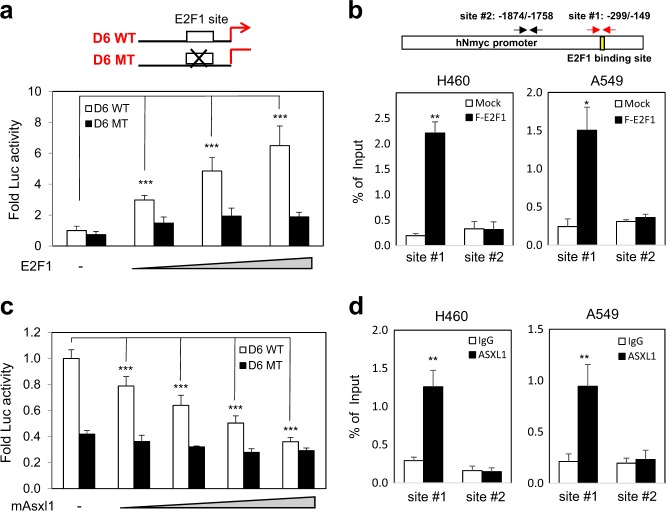
Asxl1 represses *Nmyc* expression. For quantification, three independent experiments were performed. Data are means ± SD (^**^P < 0.01, ^***^P < 0.001). **a** Effect of E2F1 on *Nmyc* promoter activation. The WT (D6 WT) and mutant (D6 MT) E2F1-responsive sites in the Nmyc promoter are indicated. HEK293T cells were transfected with an *Nmyc* promoter-driven luciferase reporter gene and E2F1 expression vector (0, 0.01, 0.05, or 0.1 μg). **b** E2F1 binding to the E2F1-responsive site in the *Nmyc* promoter. Schematic depiction of primer pairs to map the E2F1-binding site (shown by yellow). ChIP assay using an anti-Flag antibody and primer set for the human *Nmyc* promoter in H460 and A549 cells transfected with Flag-E2F1. Chromatin binding is shown as a percentage of the input. **c** Effect of Asxl1 on *Nmyc* promoter repression. HEK293T cells were transfected with the WT or mutant *Nmyc* luciferase reporter gene and ASXL1 (0.2, 0.4, 0.6, and 0.8 μg). **d** ASXL1 binding to the *Nmyc* promoter. ChIP-qPCR was performed using an anti-ASXL1 antibody and primer set for *Nmyc* promoter in A549 and H460 cells

Given that E2f1 target genes are upregulated by *Asxl1* disruption (Fig. 4a) and *Nmyc* is a target of E2F1, we determined whether ASXL1 inhibits *Nmyc* expression via the E2F1-binding site in the *Nmyc* promoter/enhancer. To answer this question, we generated luciferase reporter plasmids containing the WT and a mutant E2F1-binding site (Supplementary Figure S5a). As shown by luciferase assay using HEK293T cells transfected with Flag-tagged E2F1, the mutant with a defective E2F1-binding site failed to mediate an E2F1 response (Fig. 5a). To confirm whether E2F1 binds to putative binding site located in the region between −233 and −149, chromatin immunoprecipitation (ChIP) assays were performed using primers specific for E2F1-binding site (site #1) and covering negative control region (site #2). We observed that the site #1 is occupied by Flag-tagged E2F1 in two human non–small-cell lung cancer cell lines, H460 and A549 (Fig. 5b). As expected, Asxl1 overexpression reduced the luciferase activity in a dose-dependent manner (Supplementary Figure S5b). Further, we demonstrated that the intact E2F1-binding site is required for ASXL1-mediated repression (Fig. 5c), likely through ASXL1 binding to the site (Fig. 5d). ASXL1 occupancy of the E2F1-responsive sites was further confirmed using three other E2F1 target genes (*CDC6*, *CCNA2*, and *CDC25A*) in two lung cell lines (Supplementary Figure S5c, d).

### ASXL1 forms a complex with host cell factor (HCF)-1, a known binding partner of E2F1

**Fig. 6:**
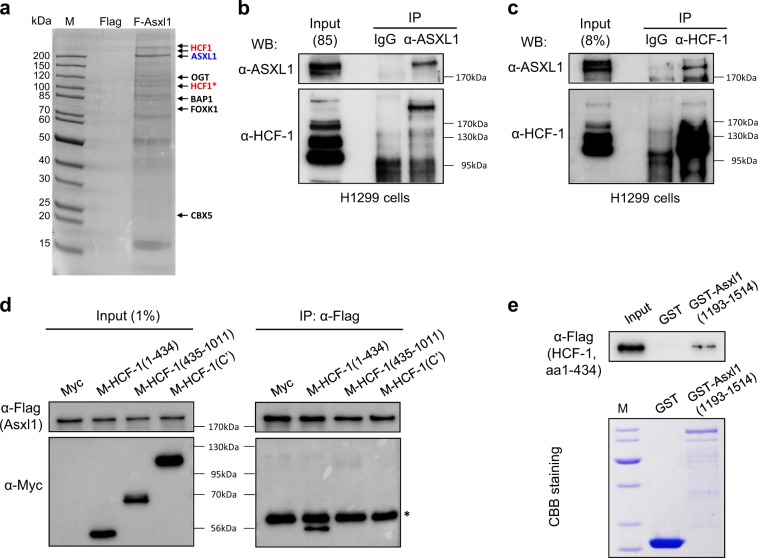
Interaction between ASXL1 and HCF-1. **a** Affinity purification of the ASXL1-bound complex. HEK293 cells stably expressing Flag-tagged murine Asxl1 (F-Asxl1) were subjected to IP using anti-Flag antibody-coupled beads. Affinity-purified ASXL1 complex proteins were separated by SDS–PAGE and stained with Coomassie Blue. Proteins identified by LC–MS/MS are indicated at right. **b**, **c** Endogenous interaction between ASXL1 and HCF-1 in H1299 cells. Reciprocal IP using an anti-ASXL1 antibody (**b**) and anti-HCF-1 antibody (**c**) was followed by WB using the indicated antibodies. **d** Mapping of the HCF-1 domain responsible for ASXL1 binding. HEK293T cells were transfected with plasmids expressing Flag-Asxl1 and Myc-tagged truncation mutants of HCF-1 (aa1–434, 435–1011, and C’, aa1436–2035). IP using an anti-Flag antibody was followed by WB using an anti-Myc antibody. Asterisk indicates IgG heavy chain. **e** Interaction between ASXL1 and HCF-1 in vitro. GST pull-down assay was performed using GST-Asxl1 (aa1193–1514) and His-tagged HCF-1N (aa1–434) protein produced by in vitro translation. A Coomassie Blue-stained gel is shown

To assess how ASXL1 mediates transcriptional repression of E2F1 target genes, including *Nmyc*, we identified ASXL1-interacting proteins. For this purpose, human HEK293T cells were engineered to overexpress Flag-tagged Asxl1. Affinity-purified Asxl1 complex using an anti-Flag antibody was identified by liquid chromatography–tandem mass spectrometry analysis (Supplementary Table S4). Major binding partners, such as HCF-1, OGT, BAP1, FOXK1, and CBX5, were indicated in the Coomassie Blue-stained gel (Fig. 6a). Of these proteins, we focused on HCF-1 as it binds to E2F1^32^, and ASXL1 failed to bind to the E2F family (data not shown). Recently, HCF-1 was identified as an ASXL1-associated protein by affinity and size-exclusion chromatography^23^. However, it is unclear whether the interaction is direct and which portion is responsible for the interaction. The endogenous interaction between ASXL1 and HCF-1 in H1299 cells was demonstrated by two reciprocal co-immunoprecipitation (co-IP) assays followed by WB performed using antibodies specific for ASXL1 and HCF-1 (Fig. 6b, c). Additionally, the binding results were confirmed in E18.5 lung tissue and A549 cells, suggesting that this interaction is not cell-line specific (Supplementary Figures S6a, b). The functional domains of mAsxl1 and hHCF-1 are depicted in Supplementary Figures S6c, d. HCF-1 protein forms a heterodimer with HCF-1N and HCF-1C, cleaved products of the full-length precursor. To map the interaction region of HCF-1 with ASXL1, we generated three HCF-1 fragments with a Myc tag: amino acids (aa) 1–434, 435–1011, and 1436–2035. After transfection with Flag-Asxl1 into HEK293T cells, cell extracts were subjected to co-IP using an anti-Flag antibody. Subsequent WB using anti-Myc antibody indicated that the N-terminal portion containing the Kelch domain of HCF-1 is responsible for its interaction with Asxl1 (Fig. 6d). Reciprocal mapping using GFP-tagged Asxl1 fragments and Flag-HCF-1 (aa1–434) showed that the C-terminal region (aa1193–1514), including the plant homeodomain (PHD) motif, is sufficient for the interaction with HCF-1 (Supplementary Figure S6e). Intriguingly, further co-IP using PHD-deleted Asxl1 indicated that no PHD motif is required for the interaction (data not shown). To determine whether the interaction is direct, a GST pull-down assay was performed using purified GST-fused Asxl1 (aa1193–1514) and in vitro-translated HCF-1 (aa1–434) (Fig. 6e). These results suggest that ASXL1 directly interacts with HCF-1 through the C-terminal region and the N-terminal Kelch domain. Because HCF-1 functions as a coactivator of E2F1^32^, we next evaluated whether HCF-1 is recruited to E2F1-responsive promoters. For this purpose, we performed ChIP-qPCR using an anti-Flag antibody in HEK293T cells transiently transfected with Flag-tagged HCF-1N (aa1–434). As shown in Supplementary Figure S6f, HCF-1N was detected on all E2F1-responsive sites of three E2F1 target genes. Further, we found that HCF-1N binds to the E2F1-binding locus of the *Nmyc* promoter in H460 and A549 cells (Supplementary Figure S6g). Overall, our data suggest that HCF-1, together with ASXL1, binds to E2F1-resposive promoters, including Nmyc, resulting in transcriptional repression.

### ASXL1 promotes the enrichment of H3K27me3 at the E2F1-binding locus

**Fig. 7:**
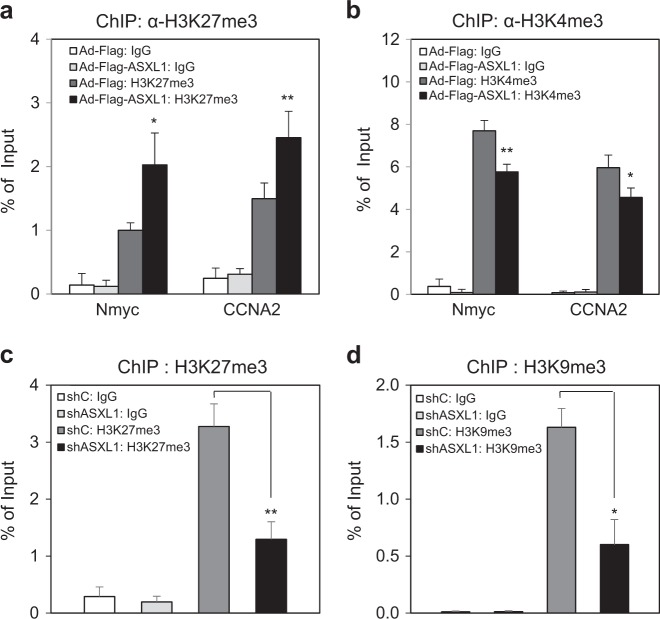
Effect of ASXL1 on epigenetic histone modifications. Verification by ChIP-qPCR under ASXL1-overexpression and -knockdown conditions. IgG was used as a control for ChIP. qPCR was performed using site #1 primer set as shown in Fig. 5. Data are means ± SD (*n* = 3, ^*^P < 0.05, ^**^P < 0.01). **a**, **b** Effect of Asxl1 overexpression on the enrichment of trimethylated histone H3 lysine 27 (H3K27me3) (**a**) and trimethylated H3 lysine 4 (H3K4me3) (**b**). A549 cells were infected with adenovirus-based Flag control or Flag-Asxl1 and subjected to ChIP-qPCR using an antibody against H3K27me3 or H3K4me3 and primer sets specific for the promoters of *Nmyc* and *CCNA2*. **c**, **d** Effect of ASXL1 depletion on the enrichment of H3K27me3 (**c**) and H3K9me3 (**d**). A549 cells were infected with adenovirus-based shLuciferase control (shC) or shASXL1 and subjected to ChIP-qPCR using an antibody against H3K27me3 or H3K9me3 and a primer set specific for the *Nmyc* promoter

Given that ASXL1 is functionally associated with either EZH2, an H3K27 methyltransferase of the PRC2 complex^7,8^, or with HP1α, a binder of methylated H3K9, for transcriptional silencing^4^, we addressed whether ASXL1, in cooperation with HCF-1, affects the enrichment of these epigenetic histone markers at the E2F1-binding locus of the *Nmyc* promoter. Upon overexpression of Asxl1 by adenoviral infection, *H3K27me3* enrichment was enhanced at the E2F1-responsive sites of the *Nmyc* and *CCNA2* promoters (Fig. 7a). In contrast, H3K4me3 enrichment was reduced on the promoters upon ASXL1 overexpression (Fig. 7b). These results were confirmed by ASXL1 depletion using adenoviral expression of shASXL1. The level of *H3K27me3* at the *Nmyc* promoter was significantly downregulated upon ASXL1 knockdown (Fig. 7c). Similarly, the promoter was less enriched in H3K9me3, another repressive histone marker (Fig. 7d), whereas H3K9ac, an active histone marker, was not affected (data not shown), thus leading to transcriptional derepression. Taken together, these data suggest that ASXL1 interacts with HCF-1 and suppresses the expression of Nmyc, an E2F1 target gene, by enriching H3K27me3 and H3K9me3 at the *Nmyc* promoter during normal fetal lung development.

## Discussion

**Fig. 8:**
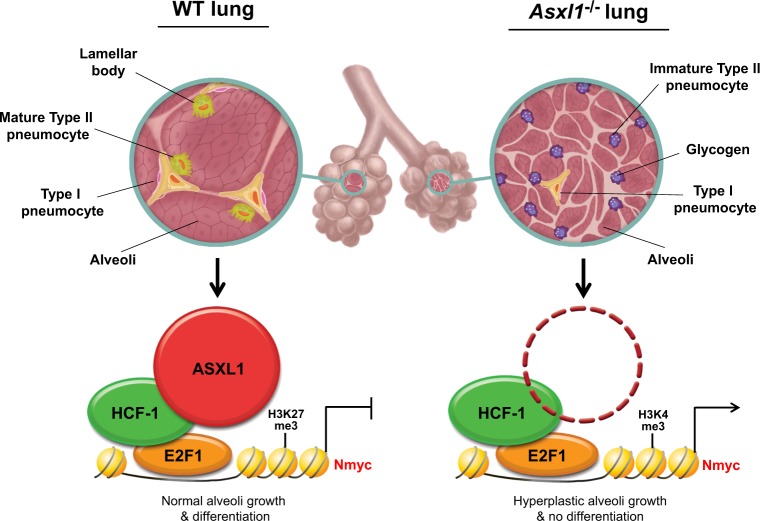
Hypothetical model for the epigenetic role of Asxl1 in regulating *Nmyc* expression during mouse fetal lung development. Details are provided in the Discussion section

In this study, we demonstrated that murine Asxl1 is indispensable for neonatal survival and plays a role in pulmonary maturation and function. Histological analyses of lungs at different gestation stages showed that branching morphogenesis is normal in *Asxl1*^−/−^ embryos; however, abnormal saccular expansion was detected at the late gestation stage. Elevated proliferation of epithelial cells in *Asxl1*-null lungs appears to be associated with dysregulated expression of Ccsp (an airway epithelial marker) and Sox9 (a maker of distal epithelial progenitor cells). Subsequent analysis of Type I and II alveolar pneumocytes revealed that *Asxl1* loss increases proliferation of immature cells but decreases differentiation of these cells for maturation (Fig. 8), which is partly consistent with previous report^22^. The reduced surfactant production and excessive glycogen content associated with defective phospholipid synthesis caused by the absence of *Asxl1* in Type II cells leads to respiratory failure and poor neonatal mortality^27^. Various factors, such as coactivator-associated arginine methyltransferase 1 (CARM1), Foxa2, Foxm1, and Mst1/2, are required for proper alveolar formation during fetal lung development^33–36^. Disruption of these genes leads to fetal lethality due to a RDS-like phenotype^37^, which includes lung collapse. As this phenotype is similar to that of the lungs of *Asxl1*^−/−^ mice, our *Asxl1*^−*/−*^ mice may represent a new model for the RDS-like phenotype. Next, lung epithelial-specific *Asxl1* conditional KO mice need to be generated by crossing with *Nkx2.1*-Cre or *Shh*-Cre mice. Additionally, analysis of ASXL1 mutations in human RDS patients by exome sequencing is warranted.

A role for ASXL1 as a tumor suppressor was suggested by the altered gene expression by Asxl1 deletion and the upregulation of genes involved in cell-cycle progression (e.g., E2F1 target genes such as *Nmyc*). The genes upregulated by *Asxl1* KO are associated with poor survival (Supplementary Figure S7a), and the genes downregulated by *Asxl1* KO are associated with good survival in lung cancer patients (Supplementary Figure S7b). Survival analysis using two lung cancer databases revealed better survival when ASXL1 expression was higher (Supplementary Figure S7c). Lung cancer patients with high levels of ASXL1 expression and lower levels of NMYC expression showed better survival (Supplementary Figure S7d), suggesting a close link between ASXl1 and NMYC. In addition to their role in progenitor cell proliferation during fetal lung development^30^, NMYC and its transcriptional regulator E2F1 are amplified and upregulated, respectively, in small-cell lung cancer^38,39^. According to our hypothesis, *Asxl1* deletion may promote progenitor cell proliferation and repress their differentiation for alveolar maturation by increasing *Nmyc* expression, leading to hyperproliferation of premature alveolar epithelial cells, which initiates lung tumorigenesis. To test this hypothesis, lung-specific conditional deletion of *Asxl1* will be required to avoid the postnatal lethality caused by constitutive knockout. In contrast to the antiproliferative role of Asxl1 in lung development, our previous study indicated that Asxl1 interacts with Akt and Ezh2 in mouse embryonic fibroblasts to maintain cell cycle progression, and *Asxl1* disruption leads to cellular senescence^8^. Therefore, we propose that ASXL1 regulates cell proliferation positively or negatively depending on the input signals during development.

A microarray analysis and subsequent validation by RT-qPCR indicated that Asxl1 represses the expression of E2F1-responsive genes, including Nmyc. To investigate the mechanism underlying this repression, we determined whether ASXL1 directly interacts with the E2F family; no interaction was detected (data not shown). BAP1 binds to E2F1-responsive gene promoters through HCF-1 and upregulates their expression, and BAP1 KO inhibits the growth of uveal melanoma cells^40,41^. However, no direct evidence of ternary complex formation by E2F1, HCF-1, and BAP1 was provided. Likewise, we identified HCF-1 as an ASXL1-binding protein and mapped the interaction sites between the C-terminal region of ASXL1 and the N-terminal Kelch domain of HCF-1 (Fig. 8). In vitro GST pull-down assays showed that the interaction is direct, not via BAP1, and this was confirmed by co-IP using a BAP1-binding-defective ASXL1 mutant (data not shown). As a transcriptional coregulator, HCF-1 associates with E2F1 and localizes to E2F1-responsive promoters to activate E2F1 by recruiting the histone H3K4 methyltransferase, mixed-lineage leukemia (MLL)^32^. Like other E2F1-responsive genes, E2F1 binds to the proximal *Nmyc* promoter and modulates its expression^29^. The role of murine Nmyc at the molecular level in fetal lung development has been mapped using mouse models and genome-wide approaches^30,31^. Thus, our studies focused on the epigenetic roles of ASXL1 and HCF-1 in the repression of E2F1 target genes, including *Nmyc* (Fig. 8). ASXL1 increased the level of the repressive histones H3K27me3 and H3K9me3 at the E2F1-responsive site. In contrast, the level of active H3K4me3 was reduced, implying that ASXL1 interacted with HCF-1 and may have interfered with its activation of E2F1 through the H3K4 methyltransferase MLL. Questions about whether ASXL1 affects the interaction between HCF-1 and MLL remain to be answered. Additionally, it will be of interest to address whether BAP1 plays a role in *Nmyc* repression by forming a complex with ASXL1 and HCF-1, and by deubiquitinating histone H2AK119 which is monoubiquitinated by PRC1. Our findings provide considerable insight into the tumor-suppressive role of ASXL1 in lung cancer and will facilitate the development of novel diagnosis and treatment strategies of pulmonary disorders including respiratory distress syndrome.

## Materials and methods

### Mouse strains and breeding

Asxl1-deficient mice (Asxl1 < tm1a(EUCOMM)Wtsi>, MGI ID:2684063, EMMA ID:03996) have been described previously^21^. All animal experiments were performed according to the regulations of the Korean Council on Animal Care, and all protocols were reviewed by the Sejong Animal Care Committee.

### Cell lines and cell culture

The HEK293T, A549, and H460 cell lines were obtained from the American Type Culture Collection (ATCC, Manassas, VA, USA). HEK293T cells were cultured in Dulbecco’s modified Eagle’s medium (DMEM) supplemented with 10% fetal bovine serum (FBS) (GenDEPOT) and an antibiotic–antimycotic (Gibco) in a 5% CO_2_ atmosphere at 37 °C. A549 and H460 cells were cultured in the RPMI-1640 medium supplemented with 10% FBS and an antibiotic-antimycotic in a 5% CO_2_ atmosphere at 37 °C.

### Plasmids and cloning

All cDNAs were constructed according to standard methods and verified by sequencing. Flag-tagged (2×) mAsxl1 and Myc-tagged hHCF-1 deletion genes were ligated into the pcDNA3 vector (Invitrogen). GFP-tagged deletion constructs were ligated into pEGFP-C3 (BD Biosciences)^21^. For GST-fused proteins, pGEX4T-1 (GE Healthcare) was used_._

### Immunohistochemistry

For immunohistochemistry, mouse embryonic lung tissues were collected in ice-cold 1× PBS and fixed in 4% paraformaldehyde overnight at 4 °C. Fixed lungs were washed with 1× PBS, gradually postfixed in 15 and 30% sucrose at 4 °C to impregnate fully, and embedded in Tissue-Tek OCT compound (Sakura Finetek, Japan). Histological analysis was performed on 6μm sections. Hematoxylin and eosin (H&E), PAS, and SBB staining were performed according to standard protocols^42,43^. Sections were processed for antigen retrieval in 0.01 M citric acid solution (pH 6.0) for 15 min. After blocking with blocking solution (5% horse serum, 3% bovine serum albumin, 0.1% Triton X-100 in 1× PBS) for 1 h, the slides were incubated with primary antibodies in blocking solution overnight at 4 °C. The primary antibodies used were Syrian hamster anti-pdpn (1:100; Santa Cruz, sc-53533), rabbit anti-proSP-C (1:200; Chemicon, 50-173-573), goat anti-CC10 (1:200; Santa Cruz, sc-9772), mouse anti-αSMA (1:300; Santa Cruz, sc-53142), mouse anti-PCNA (1:200; Santa Cruz, sc-56), and rabbit anti-SP-B (1:200; Millipore, AB3430). The slides were washed with 1× PBS and incubated with Alexa 488 goat anti-rabbit IgG at a 1:200 dilution, Alexa 568 goat anti-mouse IgG at a 1:200 dilution, Alexa 568 donkey anti-goat IgG at a 1:200 dilution, and Alexa 568 goat anti-hamster IgG at a 1:200 dilution (Invitrogen). The cover glasses were mounted using Vectashield Mounting Medium (Vector, H-1000).

### Radio and RNAscope in situ hybridization

Radio in situ hybridization (ISH) was performed as described previously^44^. Hybridization probes were generated from the subcloned pcDNA3.1 plasmids using the following primers specific for murine Asxl1: forward, *Xho*I site-ggg CTC GAG TAC AGA GTC TCA GAG CCG AC; reverse, *Bam*HI site-ggg GGA TCC GTT GCT GGA AGT GTA GT. The primers used for the murine Nmyc probe were: forward, *Xho*I site-ggg CTC GAG CCT GGG TGG CCT CAC TCC TA; reverse, *Bam*HI site-ggg GGA TCC CGC TCA AGG TAT CCT CTC CGG. For RNAscope ISH, E18.5 mice were euthanized, and their lungs frozen immediately. RNAscope is commercially available from advanced cell diagnostics (ACD). Hybridization was performed according to the RNAscope® 2.0 HD Detection kit (Brown) User Manual (ACD, #320497). In brief, lungs were embedded in optimum cutting temperature compound, and sections were thaw-mounted onto Superfrost Plus Microscope Slides (Fisher Scientific). The murine *Asxl1* (Cat No. 421961-C2) mRNA target probe was manufactured by ACD. The sections were fixed in 4% paraformaldehyde for 10 min; dehydrated in 50, 75, 95, and 100% EtOH for 5 min; and air-dried. Tissue was treated with pretreatment solution for 10 min at room temperature (RT). For RNA detection, tissue was incubated with the Asxl1 probe for 2 h at 40 °C, and the amplifier solutions were added. Each amplifier was removed by washing using washing buffer (1×) for 2 min at RT. The signals were developed with DAB with hematoxylin counterstaining. The slides were washed, mounted, and viewed under a Leica fluorescence microscope (DFC420 C).

### RNA isolation, reverse transcription, and RTqPCR

RNA isolation and qPCR were carried out as reported previously^21^ using the primers listed in Supplementary Table S5. All expression levels were normalized to that of *GAPDH*, and results were plotted as relative to WT lungs or empty controls.

### Microarray analysis

Total RNA (10 μg) isolated from E18.5 *Asxl1*^−^^/−^ and WT lungs was subjected to analysis with the Agilent Bioanalyzer System (Mouse GE 4× 44 K v2 Microarray: Agilent, Santa Clara, CA, USA) according to the manufacturer’s instructions. *Asxl1*^−/−^ signal intensities were normalized to those of the WT. Results were filtered, and the cut off was set at a twofold difference. Genes exhibiting significant differences in expression levels were classified into Gene Ontology (GO) based functional categories (http://www.geneontology.org) using the PANTHER (http://www.pantherdb.org), KEGG (http://www.genome.jp/kegg), DAVID (http://david.abcc.ncifcrf.gov), and Medline (http://www.ncbi.nlm.nig.gov) databases.

### Western blotting and co-immunoprecipitation

WB and co-IP were carried out as reported previously^21^. Briefly, lungs were homogenized in lysis buffer (50 mM Tris-Cl, pH 7.5, 150 mM NaCl, 0.5% Nonidet P-40, 5 mM EDTA, 1 mM PMSF) supplemented with protease inhibitors (Roche). Generally, 40–60 μg protein-containing supernatants were separated by electrophoresis in 10% sodium dodecyl sulfate polyacrylamide gels, transferred to nitrocellulose membranes, and incubated with the indicated primary antibodies: rabbit anti-ASXL1 (GeneTex Inc., GTX127284), anti-HCF-1 (Bethyl Laboratories, A301-399A), mouse anti-Flag (MBL International, FLA-1), mouse anti-Myc (MBL International, My3), mouse anti-GFP (Santa Cruz, sc-9996), and mouse anti-β-actin (Santa Cruz, sc-47778). The blots were incubated with peroxidase-conjugated secondary antibodies (goat anti-rabbit IgG, goat anti-mouse IgG, and goat anti-Syrian hamster IgG, as appropriate; Santa Cruz). The protein bands were detected using ECL reagent (Intron) followed by exposure to X-ray film (Agfa) or detection in a Vilber Fusion Solo 2 Chemiluminescence System (Fisher Biotec). For IP, we used a rabbit anti-ASXL1 polyclonal antibody raised against aa 233–247 of mouse Asxl1^3^.

### Construction of the Nmyc promoter-luciferase reporter

The *Nmyc* promoter (−3.2 kb in length) generated by mouse tail gDNA was subcloned upstream of a luciferase reporter gene into the pGL2-basic vector (Promega). The 5′-deletion and E2F1-binding site mutants in the *Nmyc* promoter were produced by PCR. Primers were designed with *Nhe*l and *Bam*Hl sites at the ends of the products (see Supplementary Table S5). All plasmids were verified by restriction enzyme digestion and DNA sequencing. Luciferase activity was measured as described previously^3^.

### Recombinant adenovirus

For overexpression in mammalian cells, Asxl1 adenovirus was generated using the pAdEasy system^45^. Flag-tagged mAsxl1 was subcloned into the pAdTrackCMV plasmid and recombined with the pAdEasy adenovirus backbone plasmid in *E*. *coli* BJ5183. QBI-293A cells were transfected with recombinant plasmid, and the virus was amplified. The virus titer was measured using an Adeno-X Rapid Titer Kit (Clontech). For KO in mammalian cells, recombinant adenovirus-expressing shRNA was prepared. The duplex DNA effective for both human and mouse ASXL1 (Supplementary Table S5) was digested with *Not*l and subcloned into the digested PBS/U6 vector (Addgene). The U6 promoter-driven shASXL1 was excised and ligated into the pAdtrack vector (Addgene). The pAdTrack-U6 shASXL1 obtained was recombined with pAdEasy-1 by transformation in *E. coli* BJ5183. Recombinant adenovirus was produced by transfecting the recombinant plasmid into QBI-293A cells. Infection and KO efficiency were monitored by GFP fluorescence and RT-qPCR, respectively. To evaluate the optimal multiplicity of infection (MOI) for maximal infection and transgene expression, 60-mm dishes containing 2×10^5^ A549 cells were infected with adenovirus at MOIs of 50, 100, and 200 for 24 h. Adenoviral infection efficiency was assessed based on GFP expression. *Asxl1* expression in A549 cells was evaluated by RT-PCR and WB.

### Glutathione S-transferase pull-down assay

Glutathione S-transferase (GST) pull-down assay was performed as described previously^3^. GST-fused Asxl1 (aa1193–1514) protein was expressed in *E. coli* and purified using glutathione-Sepharose beads (GE Healthcare). Flag-HCF-1 (aa1–434) protein was translated in vitro using a TNT^®^ rabbit reticulocyte system (Promega). Briefly, 2 μg of GST-Asxl1 (or GST) was incubated with 10μL of Flag-HCF-1 protein. Bound protein was visualized by WB using an anti-Flag M2 monoclonal antibody (Sigma, F-3165).

### Chromatin immunoprecipitation

ChIP assay was performed as described previously with some modifications^46^. A549 and H460 cells were transfected with the Flag-E2F1 expression plasmid. Cross-linked sheared chromatin complexes were recovered by IP using the antibodies indicated, and the cross-linking was reversed. The released DNA pellets were amplified by quantitative PCR using a primer pair that encompassed the E2F1-binding sites in the *Nmyc*, *CDC6*, *CCNA2*, and *CDC25A* promoter regions (Supplementary Table S5). Antibodies used for histone modifications are mouse anti-H3K4me3 (Millipore, 07-473), mouse anti-H3K9me3 (Millipore, 07-442), mouse anti-H3K27me3 (Abcam, ab6002; Millipore, 07-449).

### TUNEL assay

In situ cell death was evaluated using a TUNEL assay kit (Roche). Lungs were fixed, permeabilized, and incubated in TUNEL reagent micro-drops comprising 10% of the enzymatic solution (deoxynucleotidyl terminal transferase) and 90% of the marking solution (2′-deoxyuridine 5′-triphosphate-DUTP + fluorescein  isothiocyanate-conjugated-FITC) for 1 h in a humid chamber at 37 °C in the dark. The positive control was a sample treated with 1 IU/μL DNase (Promega); the negative control was incubated in micro-drops containing only marking solution. After washing, samples were stained with Hoechst 33342 (Sigma), and the cover glasses were mounted with Vectashield Mounting Medium (Vector, H-1000).

### Purification of ASXL1-associated protein complex

HEK293 cells stably expressing Flag-tagged Asxl1 or Flag vector were grown in 20×150 cm dishes. Affinity purification of the ASXL1 protein complex was carried out as reported previously^44^.

### Statistical analysis

Data are means ± standard deviation of at least three independent experiments. Comparisons between multiple groups were performed by paired Student’s *t* tests. *P* values < 0.05 (^*^), 0.01 (^**^), or 0.001 (^***^) were considered to indicate statistical significance.

## Electronic supplementary material

Supplementary Figure legends

Supplementary Figures 1–7

Supplementary Table S1

Supplementary Table S2

Supplementary Table S3

Supplementary Table S4

Supplementary Table S5

supplementary figure legends
