# Supplementary material for: Asxl1 exerts an antiproliferative effect on mouse lung maturation via epigenetic repression of the E2f1-Nmyc axis
Source: Cell Death Dis. 2018 Nov 2;9(11):1118. doi: 10.1038/s41419-018-1171-z (PMC6215009; doi:10.1038/s41419-018-1171-z)
Supplement: Supplementary file 1 — Supplementary Figure legends [file 41419_2018_1171_MOESM1_ESM.docx]

**Supplementary Figure Legends**

**Supplementary Figure S1.** Phenotype and postnatal lethality of *Asxl1*-deleted mice. (**a**) Size difference between wild-type (WT) and *Asxl1*-null mice at postnatal day 0 (P0). (**b**) Expression of Asxl1 protein in mouse lung. Lysates from WT and *Asxl1*^-/-^ lungs at E18.5 were subjected to Western blotting (WB) using an anti-Asxl1 antibody. Asterisks indicate non‑specific bands. (**c**) Asxl1 mRNA level in mouse lung by RT-qPCR. Data are means ± SD (*n*= 11, ****P* < 0.001). (**d**) Survival rate of mice after birth. Data are means ± SD (*n*= 11, WT; *n* = 26, *Asxl1*^+/-^; *n*= 8, *Asxl1*^-/-^ mice; ***P* < 0.01). (**e**) Phenotypic differences in the lungs of WT and *Asxl1*-null mice. Scale bar, 100 μm. (**f**) Weight difference: whole body, lung, and lung/whole body. Each was decreased by 14%, 24%, and 17%, respectively, relative to the WT. Data are means ± SD (*n*= 11, WT; *n*= 26, *Asxl1*^+/-^; *n*= 8, *Asxl1*^-/-^ mice; **P* < 0.05, ***P* < 0.01, ****P*< 0.001). (**g**) Alcian Blue and Alizarin Red staining of tracheal cartilage at E18.5. (**h, i**) Hematoxylin and eosin (H&E) staining of the trachea (**h**) and heart (**i**) at E18.5. Scale bar, 100 μm. (**j**) Expression of *Asxl1* in E18.5 lung. Sagittal sections were made, and the Asxl1 mRNA distribution was visualized by ISH using a radioisotope. Red indicates the lung. Box indicates a horizontal section of an isolated lung. (**k**) X-gal staining of *Asxl1*^-/-^ lung at E14.5, E16.5, E18.5, and P0. (**l**) Isolation of lung epithelial and mesenchymal cells at E18.5 according to the protocol reported.^1^ (**m**) Expression of Asxl1 at E18.5. Expression was monitored by RT-qPCR using epithelial marker Ccsp and mesenchymal marker Vimentin (Forward: GAT CAG CTC ACC AAC GAC / Reverse: AAG ACG TGC CAG AGA AGC AT). Data are normalized to GAPDH.

**Supplementary Figure S2.** Increased proliferation of distal and proximal pulmonary cells in *Asxl1*^-/-^ lung. (**a**, **b**) Evaluation of cell proliferation by Ki-67 immunofluorescence (**a**) and quantification of Ki-67 positive cells (**b**). (**c**, **d**) Evaluation of apoptosis by TUNEL assay (**c**) and quantification of TUNEL-positive cells (**d**). Scale bar, 100 μm. Data are means ± SD (*n*= 4, WT mice; *n*= 4, Asxl1^-/-^ mice; **P* < 0.05). (**e**) Co-expression of PCNA and Nkx2.1. Co-immunostaining was performed using antibodies against PCNA (Santa Cruz, sc-56) and Nkx2.1 (Abcam, ab76013). (**f**) Expression of Ccsp mRNA at P0. Monitored by RT-qPCR (**P < 0.05)*. (**g**, **h**) Expression of Sox9 at E18.5 evaluated by immunostaining (**g**) and RT-qPCR (**h**). (**I**, **j**) Ex vivo lung culture at E10.5 (**i**) and E12.5 (**j**). Organ culture was performed as reported previously.^2,3^

**Supplementary Figure S3.** Increased PAS staining in *Asxl1*-deleted mouse lung. (**a**) Evaluation of distal and proximal PAS-positive cells by PAS staining in sections of WT and *Asxl1^-/-^* E18.5 lungs. (**b**) Quantification of PAS-positive cells. Scale bar, 100 μm. Data are means ± SD (*n*= 6, WT mice; *n*= 6, Asxl1^-/-^ mice; **P < 0.05,* ***P* < 0.01).

**Supplementary Figure S4.** Upregulation of genes associated with cell proliferation in *Asxl1*^-/-^ lung. For mRNA quantification, three RT-qPCRs were independently performed. Data are means ± SD (**P < 0.05,* ***P* < 0.01, ****P* < 0.001). (**a**) Gene Ontology (GO) analysis. Pie chart and bar graph show GO for genes differentially expressed according to biological process. (**b**, **c**) Gene Set Enrichment Analysis was performed by comparing *Asxl1*-responsive genes with two gene sets associated with cell proliferation (**b**; CELL_PROLIFERATION_GO_ 0008283) and E2F signaling (**c**; E2F_TARGET_GENES). (**d**) Validation of mRNA levels. E2F1 target genes were analyzed by RT-qPCR. (**e**) Effect of ASXL1 knockdown on the expression of E2F1 target genes. A549 cells were infected with control shRNA (shC) or ASXL1-specific shRNA (shASXL1). Total RNA was isolated and subjected to RT-qPCR using primer sets specific to ASXL1 and the indicated E2F1 target genes. (**f**) Effect of ASXL1 overexpression on the expression of E2F1 target genes. A549 cells were infected using an adenovirus-based FLAG-Asxl1 expression system (Ad-Flag-Asxl1) for RT-qPCR analysis. (**g**) GSEA using the WEI_MYCN_TARGETS_WITH_E_ BOX gene set. (**h**) Effect of Asxl1 on the expression of surfactant genes and type II cell markers (*Sftpa, Sftpb, and Sftpc*) during fetal lung development.

**Supplementary Figure S5.** Effect of ASXL1 on the expression of E2F1 target genes. All quantitative assays were performed three times. Data are means ± SD (*n*= 3, ***P* < 0.01, ****P* < 0.001). (**a**) Schematic of the murine *Nmyc* promoter (-3.2kb) and -735 bp fragment with wild-type (D6 WT) or mutant (D6 MT) E2F1-binding sites. Two point mutations were introduced into the potential E2F1 binding sequence to generate a mutant. (**b**) Repression by Asxl1 of *Nmyc* promoter-driven luciferase reporter activation. HEK293 cells were transfected with an *Nmyc* promoter-driven luciferase reporter gene and Flag-mAsxl1 (0.2, 0.4, and 0.8 μg). Lysates were subjected to luciferase (LUC) assays. (**c**, **d**) ASXL1 binding to the E2F1-responsive sites in A549 cells (**c**) and H460 cells (**d**). ChIP-qPCR assays were performed using an anti-ASXL1 antibody and primer sets for the indicated genes. IgG was used as a negative control.

**Supplementary Figure S6.** Mapping the regions responsible for the interaction between ASXL1 and HCF-1. (**a**, **b**) Endogenous interaction between ASXL1 and HCF-1 in mouse lung at E18.5 (**a**) and human A549 cells (**b**). IP using an anti-ASXL1 antibody was followed by WB using anti-ASXL1 and -HCF-1 antibodies. (**c**, **d**) Schematic representation of mouse Asxl1 (**c**) and human HCF-1 (**d**). Fragments used for domain mapping. (**e**) Mapping of the Asxl1 domain responsible for HCF-1 binding. HEK293T cells were transfected with plasmids expressing FLAG-HCF-1N (aa 1–434) and GFP-tagged various truncations of mAsxl1 (aa 1–370, 371–655, 656–1,192, and 1,193–1,514). IP using an anti-FLAG antibody was followed by WB using an anti-GFP antibody. Asterisk indicates a nonspecific band or IgG heavy chain. (**f**) HCF-1 binding to the E2F1-responsive sites. A549 cells were transfected with FLAG-HCF-1 (aa1-434) and subjected to ChIP-qPCR using an anti-FLAG antibody and primer sets for the indicated genes. (**g**) HCF-1 binding to the E2F1-responsive site in the *Nmyc* promoter. ChIP-qPCR was performed using primers for the *Nmyc* promoter in H460 and A549 cells. Data are means ± SD (*n*= 3, ***P* < 0.01, ****P* < 0.001).

**Supplementary Figure S7.** Potential role of ASXL1 in lung cancer and patient survival. (**a**, **b**) Gene set enrichment analysis was performed by comparing *Asxl1*-responsive genes with two gene sets associated with the survival of patients with lung cancer (**a**, SHEDDEN_ LUNG_CANCER_POOR_SURVIVAL_A6; **b**, SHEDDEN_LUNG_CANCER_GOOD_ SURVIVAL_A4). (**c**, **d**) Survival of lung cancer patients was analyzed according to ASXL1 (c) and NMYC (**d**) expression using the Gene Expression Omnibus (GEO) databases GSE3141 and GSE31210.

**References**

1. Im, S. K. et al. Disruption of Sorting Nexin 5 Causes Respiratory Failure Associated with Undifferentiated Alveolar Epithelial Type l cells in mice. *PLos One* **8**, e58511 (2013).
2. Warburton, D. et al. Epigenetic role of epidermal growth factor expression and signalling in embryonic mouse Lung morphogenesis. *Dev Biol*. **149**, 123-133 (1992).
3. Carraro, G. et al. Mouse embryonic lung culture, a system to evaluate the molecular mechanisms of branching. *J. Vis. Exp*. **40**, 2035 (2010).
