# Supplementary material for: Asxl1 exerts an antiproliferative effect on mouse lung maturation via epigenetic repression of the E2f1-Nmyc axis
Source: Cell Death Dis. 2018 Nov 2;9(11):1118. doi: 10.1038/s41419-018-1171-z (PMC6215009; doi:10.1038/s41419-018-1171-z)
Supplement: Supplementary file 2 — Supplementary Figures 1–7 [file 41419_2018_1171_MOESM2_ESM.pptx]

## Slide 1
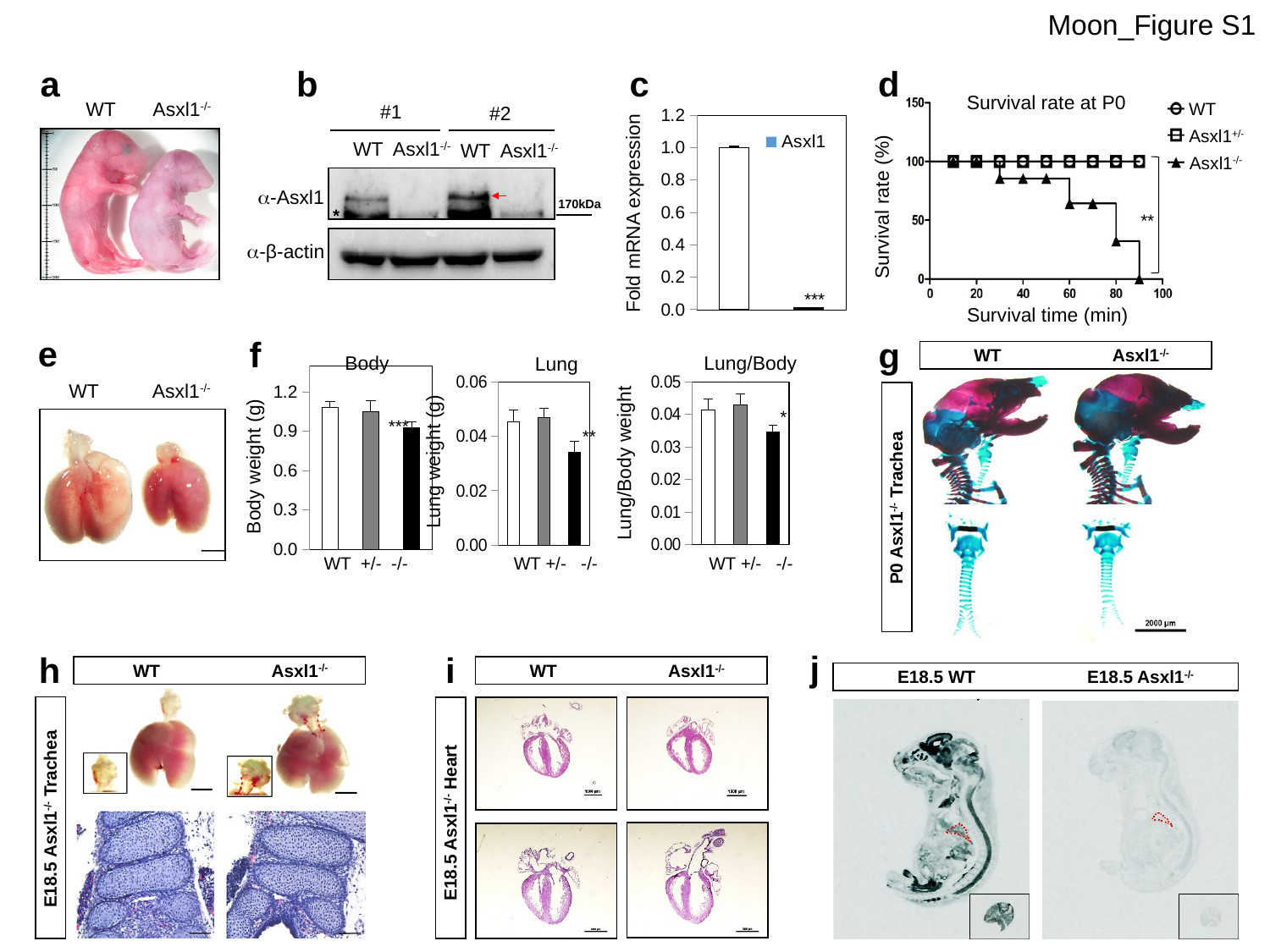

Moon_Figure S1
a
c
d
b
Survival rate at P0
 WT Asxl1-/-
WT
#1
#2
 WT Asxl1-/-
 WT Asxl1-/-
a-Asxl1
170kDa
*
a-β-actin
### Chart
| Category | Asxl1 |
|---|---|
| WT | 1.0 |
| Asxl1-/- | 0.010726829091959067 |Fold mRNA expression
Asxl1+/-
Survival rate (%)
Asxl1-/-
**
***
Survival time (min)
e
f
g
WT Asxl1-/-
 P0 Asxl1-/- Trachea
Body
Lung/Body
Lung
### Chart
| Category | |
|---|---|
| WT | 0.045250000000000005 |
| Asxl1+/- | 0.04701333333333333 |
| Asxl1-/- | 0.03423333333333333 |
### Chart
| Category | |
|---|---|
| WT | 1.0823272727272728 |
| Asxl1+/- | 1.0474038461538462 |
| Asxl1-/- | 0.9278249999999999 |
### Chart
| Category | |
|---|---|
| WT | 0.04132249818125065 |
| Asxl1+/- | 0.04293876996330275 |
| Asxl1-/- | 0.03449429941324772 |WT Asxl1-/-
Lung/Body weight
Lung weight (g)
Body weight (g)
*
***
**
WT +/- -/-
WT +/- -/-
WT +/- -/-
j
h
WT Asxl1-/-
 E18.5 Asxl1-/- Trachea
i
WT Asxl1-/-
 E18.5 Asxl1-/- Heart
E18.5 WT E18.5 Asxl1-/-

## Slide 2
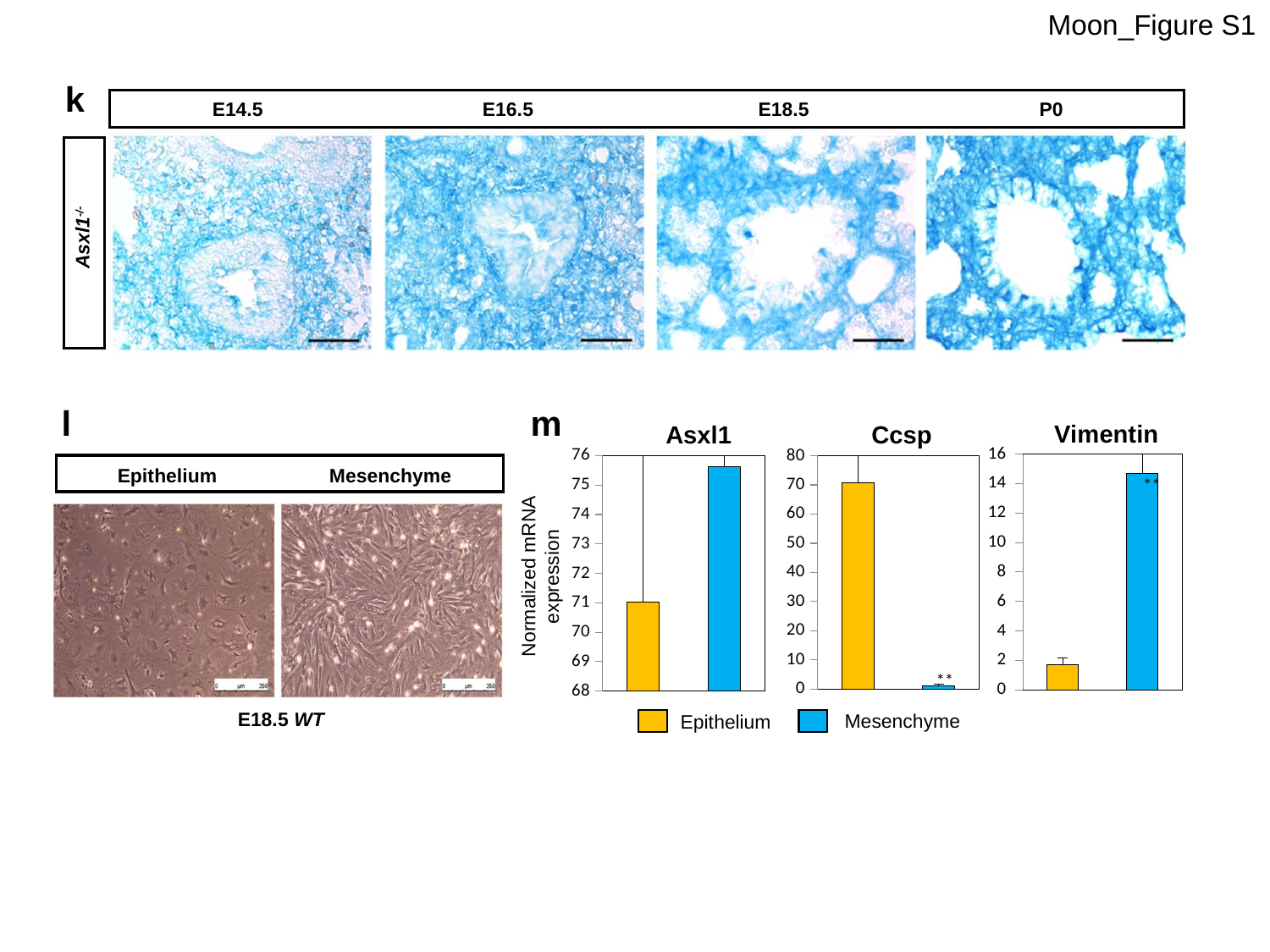

Moon_Figure S1
k
E14.5 E16.5 E18.5 P0
Asxl1-/-
m
l
Vimentin
Asxl1
Ccsp
### Chart
| Category | ccsp |
|---|---|
| Mesenchyme | 70.79592124707717 |
| Epithelial | 1.2439983021379237 |
### Chart
| Category | |
|---|---|
### Chart
| Category | |
|---|---|Normalized mRNA expression
Epithelium
Mesenchyme
**
**
E18.5 WT
Mesenchyme
Epithelium

## Slide 3
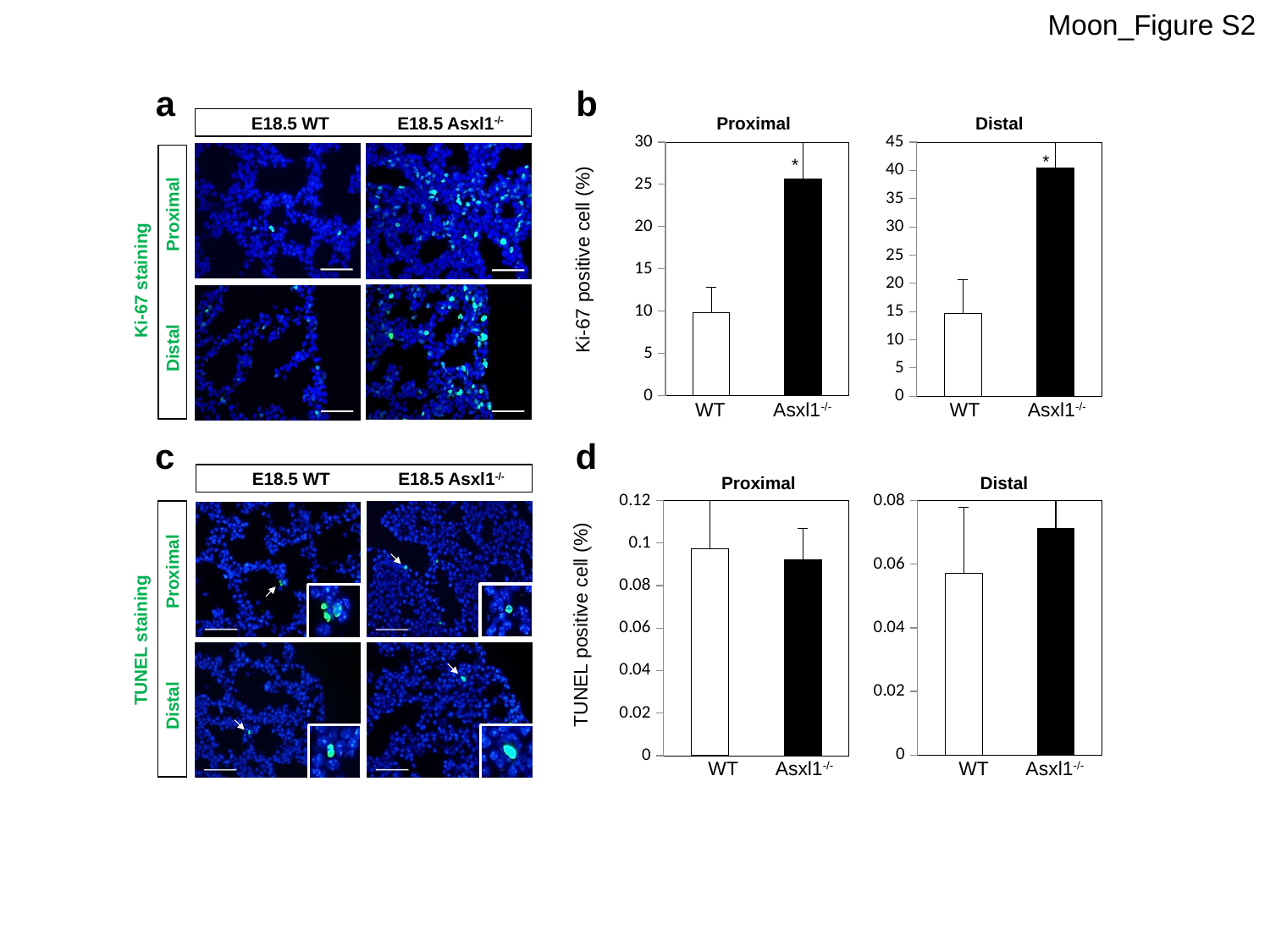

Moon_Figure S2
b
a
E18.5 WT E18.5 Asxl1-/-
Proximal Distal
### Chart
| Category | Lung distal |
|---|---|
| WT | 14.621975165167 |
| Asxl-/- | 40.4169633609071 |
### Chart
| Category | Lung proximal |
|---|---|
| WT | 9.85627784237223 |
| Asxl-/- | 25.6391540204617 |Ki-67 positive cell (%)
*
*
Distal Proximal
Ki-67 staining
WT Asxl1-/-
WT Asxl1-/-
d
c
E18.5 WT E18.5 Asxl1-/-
Proximal Distal
### Chart
| Category | tunnel |
|---|---|
| WT | 0.0973317656737412 |
| HOMO | 0.0921517897206477 |
### Chart
| Category | ki-67 |
|---|---|
| WT | 0.057240884613697 |
| HOMO | 0.0712655638238897 |
TUNEL positive cell (%)
Distal Proximal
TUNEL staining
WT Asxl1-/-
WT Asxl1-/-

## Slide 4
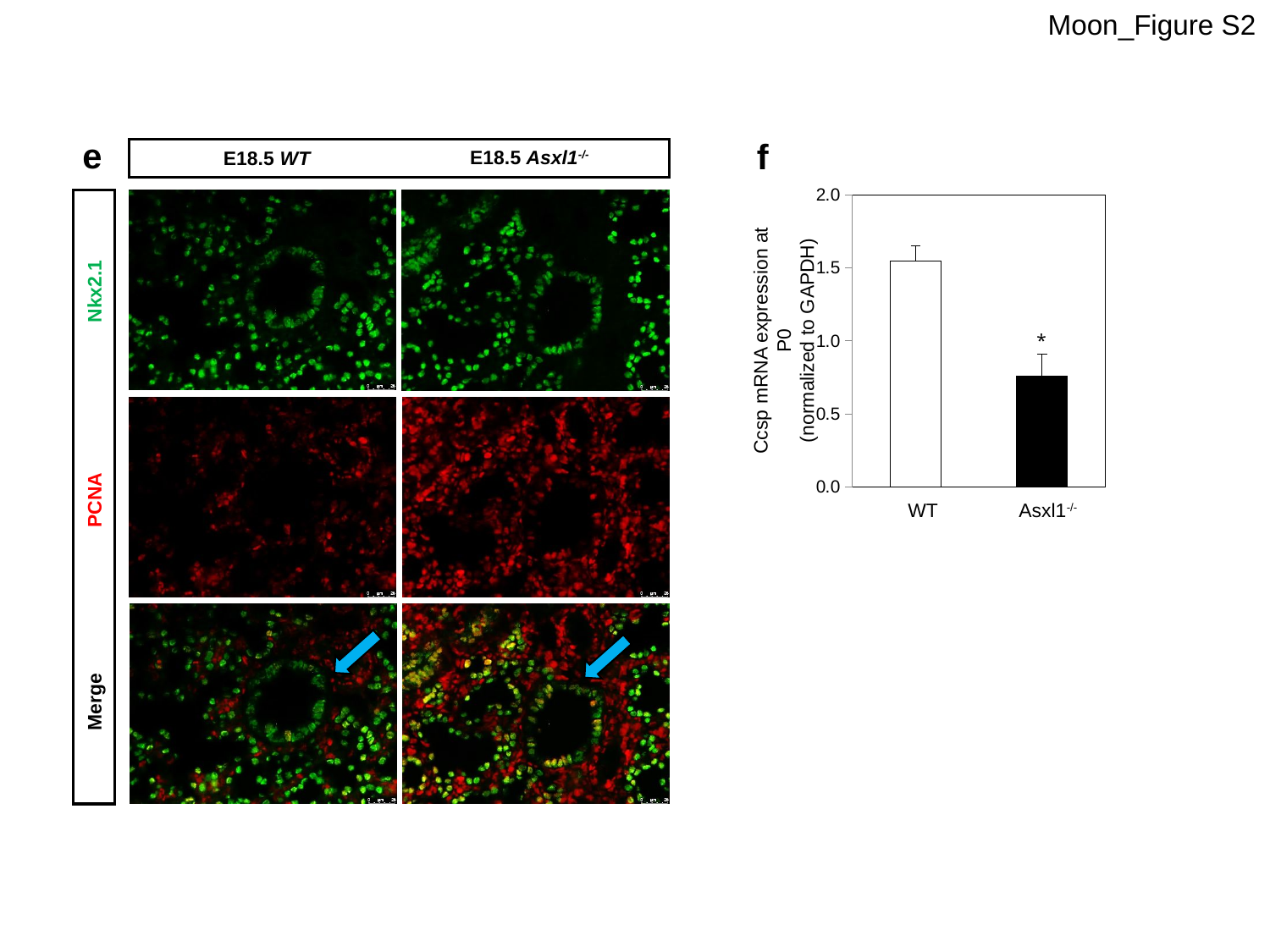

Moon_Figure S2
e
f
E18.5 Asxl1-/-
E18.5 WT
### Chart
| Category | |
|---|---|
| P0 WT | 1.5468949313701745 |
| P0 Asxl1-/- | 0.7591508432312508 |
Ccsp mRNA expression at P0
(normalized to GAPDH)
Nkx2.1
*
PCNA
WT Asxl1-/-
Merge

## Slide 5
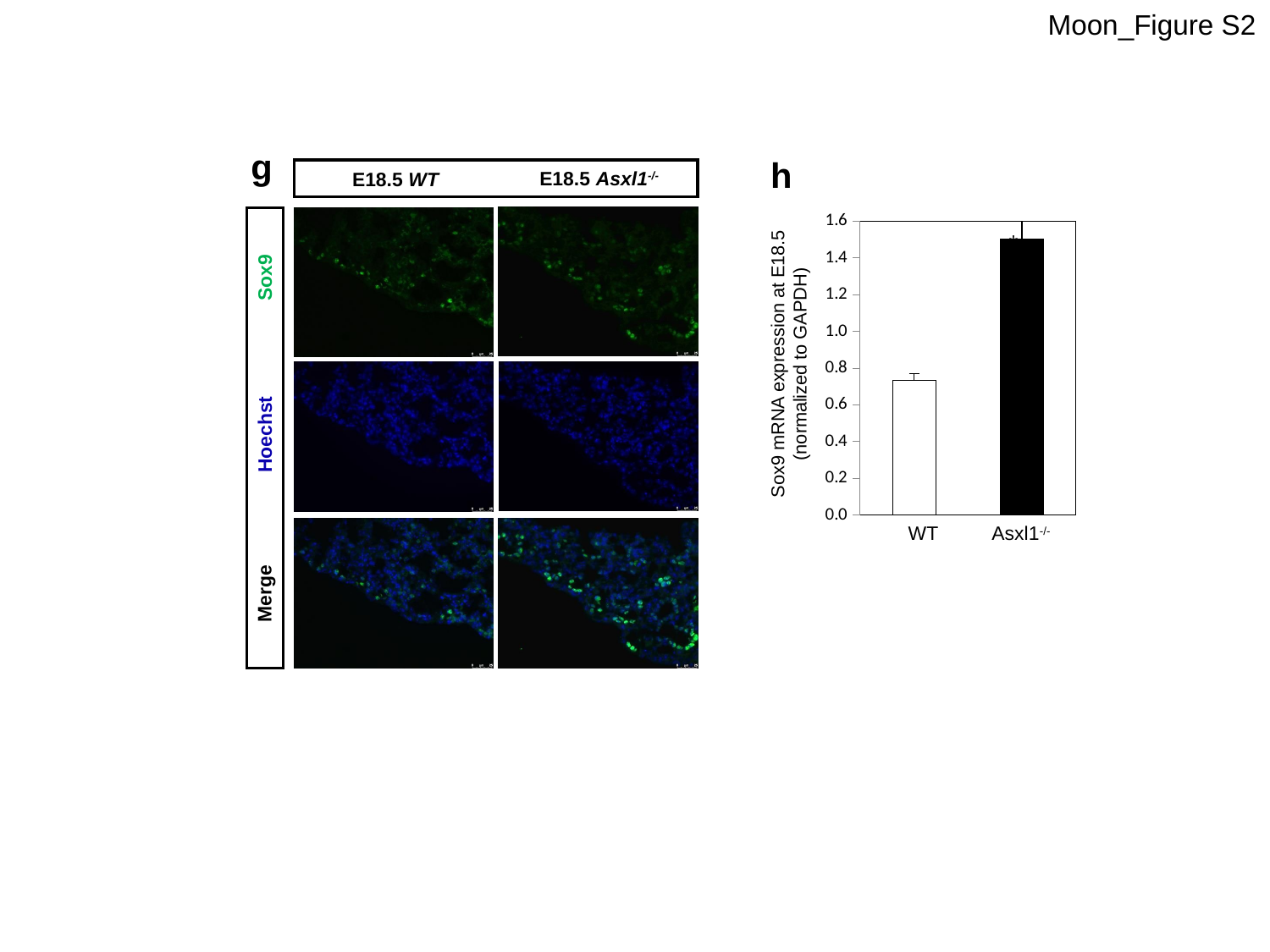

Moon_Figure S2
g
h
E18.5 Asxl1-/-
E18.5 WT
### Chart
| Category | |
|---|---|
| E18.5 WT | 0.7341970694138541 |
| E18.5 Asxl1-/- | 1.5002863414485104 |Sox9 mRNA expression at E18.5
(normalized to GAPDH)
*
WT Asxl1-/-
Sox9
Hoechst
Merge

## Slide 6
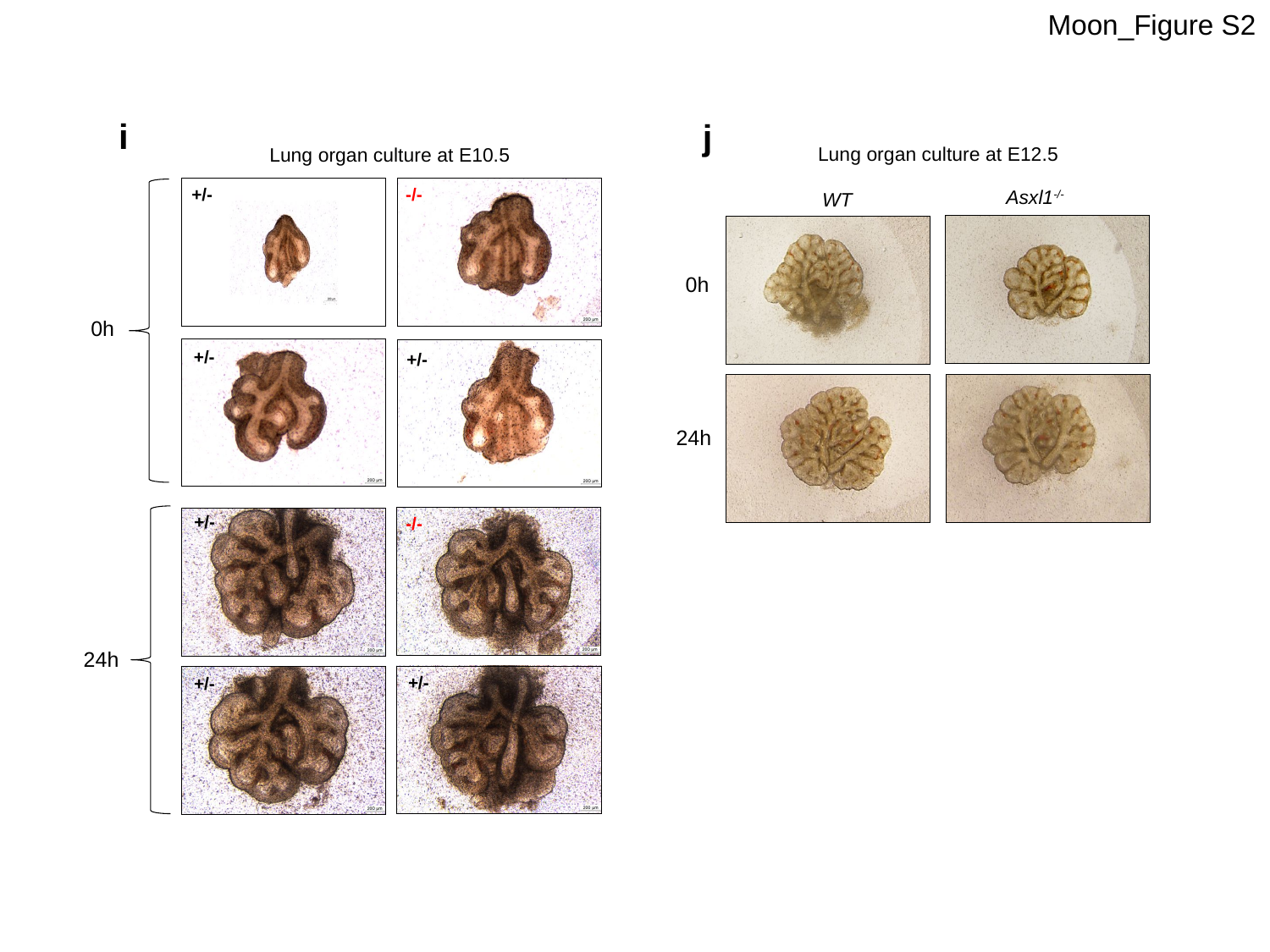

Moon_Figure S2
i
j
Lung organ culture at E12.5
Lung organ culture at E10.5
+/-
-/-
+/-
+/-
0h
Asxl1-/-
WT
0h
24h
+/-
-/-
+/-
+/-
24h

## Slide 7
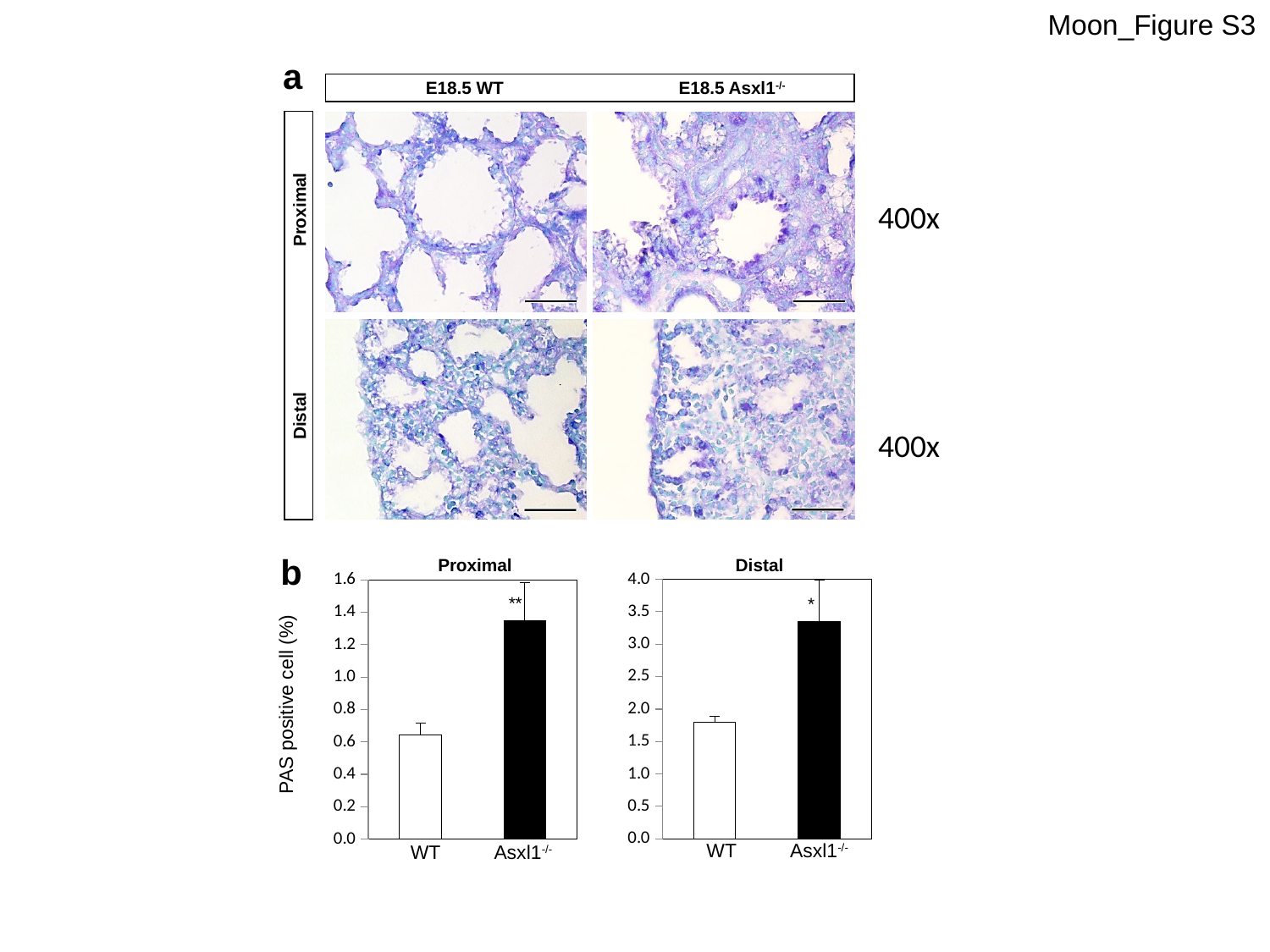

Moon_Figure S3
a
E18.5 WT E18.5 Asxl1-/-
Proximal
400x
Distal
400x
b
Proximal Distal
### Chart
| Category | |
|---|---|
| WT | 1.79984381297479 |
| Asxl1-/- | 3.34635165390017 |
### Chart
| Category | |
|---|---|
| WT | 0.642199057968189 |
| Asxl1-/- | 1.34629479792116 |PAS positive cell (%)
**
*
WT Asxl1-/-
WT Asxl1-/-

## Slide 8
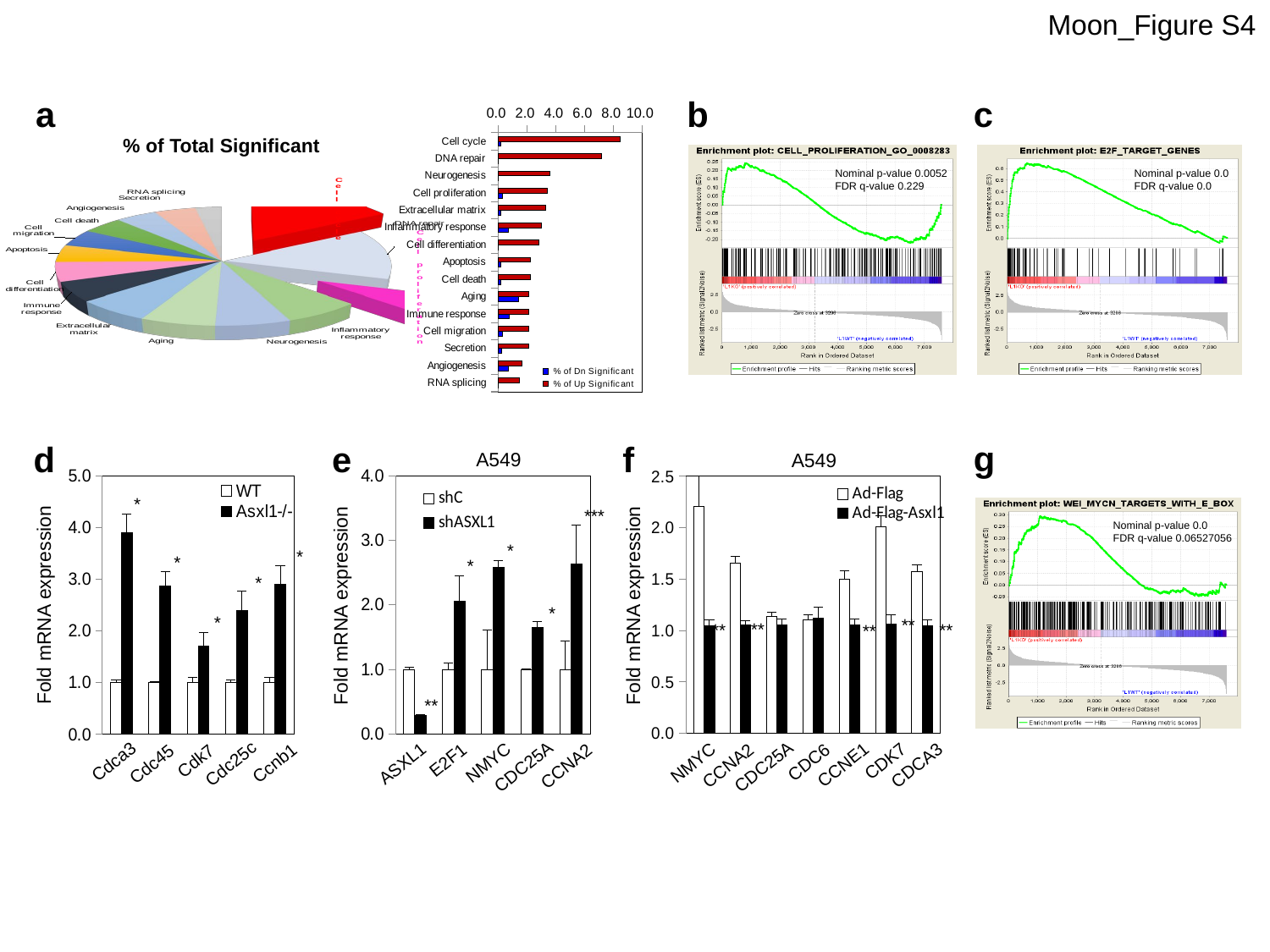

Moon_Figure S4
c
b
a
### Chart
| Category | % of Up Significant | % of Dn Significant |
|---|---|---|
| Cell cycle | 8.480325644504749 | 0.20352781546811397 |
| DNA repair | 7.192575406032482 | 0.0 |
| Neurogenesis | 3.576982892690513 | 0.0 |
| Cell proliferation | 3.409758965314521 | 0.29394473838918284 |
| Extracellular matrix | 3.285420944558522 | 0.20533880903490762 |
| Inflammatory response | 2.9885057471264367 | 0.6896551724137931 |
| Cell differentiation | 2.8223220012828736 | 0.032071840923669014 |
| Apoptosis | 2.2650056625141564 | 0.16987542468856173 |
| Cell death | 2.2233986236103758 | 0.15881418740074113 |
| Aging | 2.127659574468085 | 1.4184397163120568 |
| Immune response | 2.127659574468085 | 0.7978723404255319 |
| Cell migration | 2.1119324181626187 | 0.31678986272439286 |
| Secretion | 2.1052631578947367 | 0.23391812865497078 |
| Angiogenesis | 1.639344262295082 | 0.702576112412178 |
| RNA splicing | 1.488095238095238 | 0.0 |% of Total Significant
[unsupported chart]
Nominal p-value 0.0052
FDR q-value 0.229
Nominal p-value 0.0
FDR q-value 0.0
g
f
e
d
A549
A549
### Chart
| Category | Ad-Flag | Ad-Flag-Asxl1 |
|---|---|---|
| | 2.2094310230468053 | 1.0468770468564064 |
| | 1.659105903247904 | 1.0520076838956702 |
| | 1.1399680883554522 | 1.0577556872837492 |
| | 1.1074239383495255 | 1.1176849185015503 |
| | 1.5015883312557448 | 1.0584202111948564 |
| | 2.0131656155425763 | 1.066453829425079 |
| | 1.569922600763105 | 1.0460087497182426 |
### Chart
| Category | shC | shASXL1 |
|---|---|---|
| Asxl1 | 1.0 | 0.29272988767049224 |
| CDC45 | 1.0 | 2.057598773608836 |
| CDK7 | 1.0 | 2.5816823596336556 |
| CDC25C | 1.0 | 1.6524546772641975 |
| CCNB1 | 1.0 | 2.6294793515194512 |
### Chart
| Category | WT | Asxl1-/- |
|---|---|---|
| CDCA3 | 1.0 | 3.9077404859084064 |
| CDC45 | 1.0 | 2.865835852485813 |
| CDK7 | 1.0 | 1.7136240432611658 |
| CDC25C | 1.0 | 2.399890450677599 |
| CCNB1 | 1.0 | 2.897433591211465 |*
Fold mRNA expression
Nominal p-value 0.0
FDR q-value 0.06527056
***
Fold mRNA expression
Fold mRNA expression
*
*
*
*
*
*
*
**
**
**
**
**
**
E2F1
Cdk7
CDK7
Cdca3
CDC6
NMYC
NMYC
Ccnb1
ASXL1
Cdc25c
Cdc45
CCNE1
CDCA3
CCNA2
CCNA2
CDC25A
CDC25A

## Slide 9
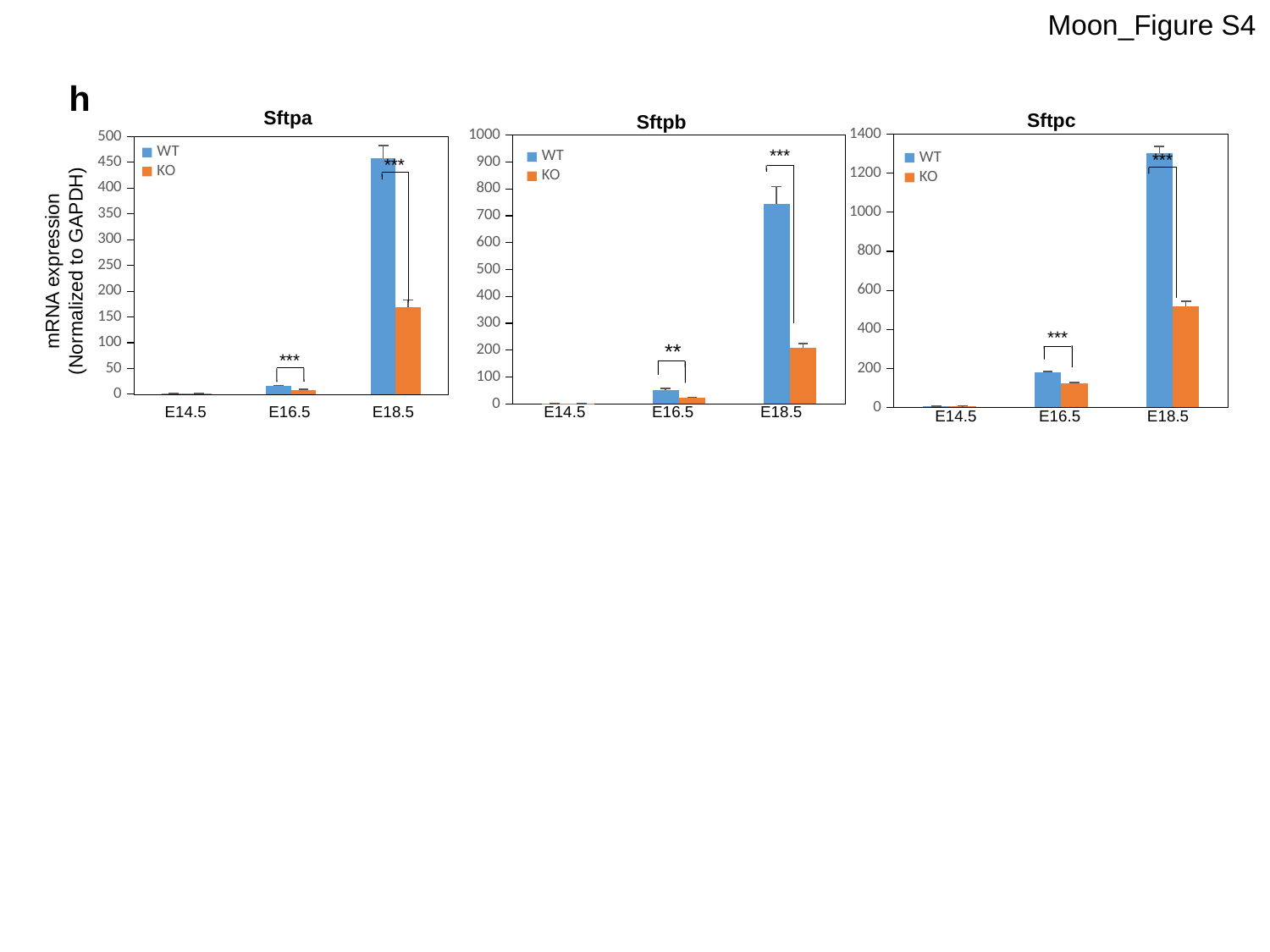

Moon_Figure S4
h
Sftpa
Sftpc
Sftpb
### Chart
| Category | WT | KO |
|---|---|---|
### Chart
| Category | WT | KO |
|---|---|---|
### Chart
| Category | WT | KO |
|---|---|---|***
***
***
mRNA expression
(Normalized to GAPDH)
***
**
***
E14.5 E16.5 E18.5
 E14.5 E16.5 E18.5
 E14.5 E16.5 E18.5

## Slide 10
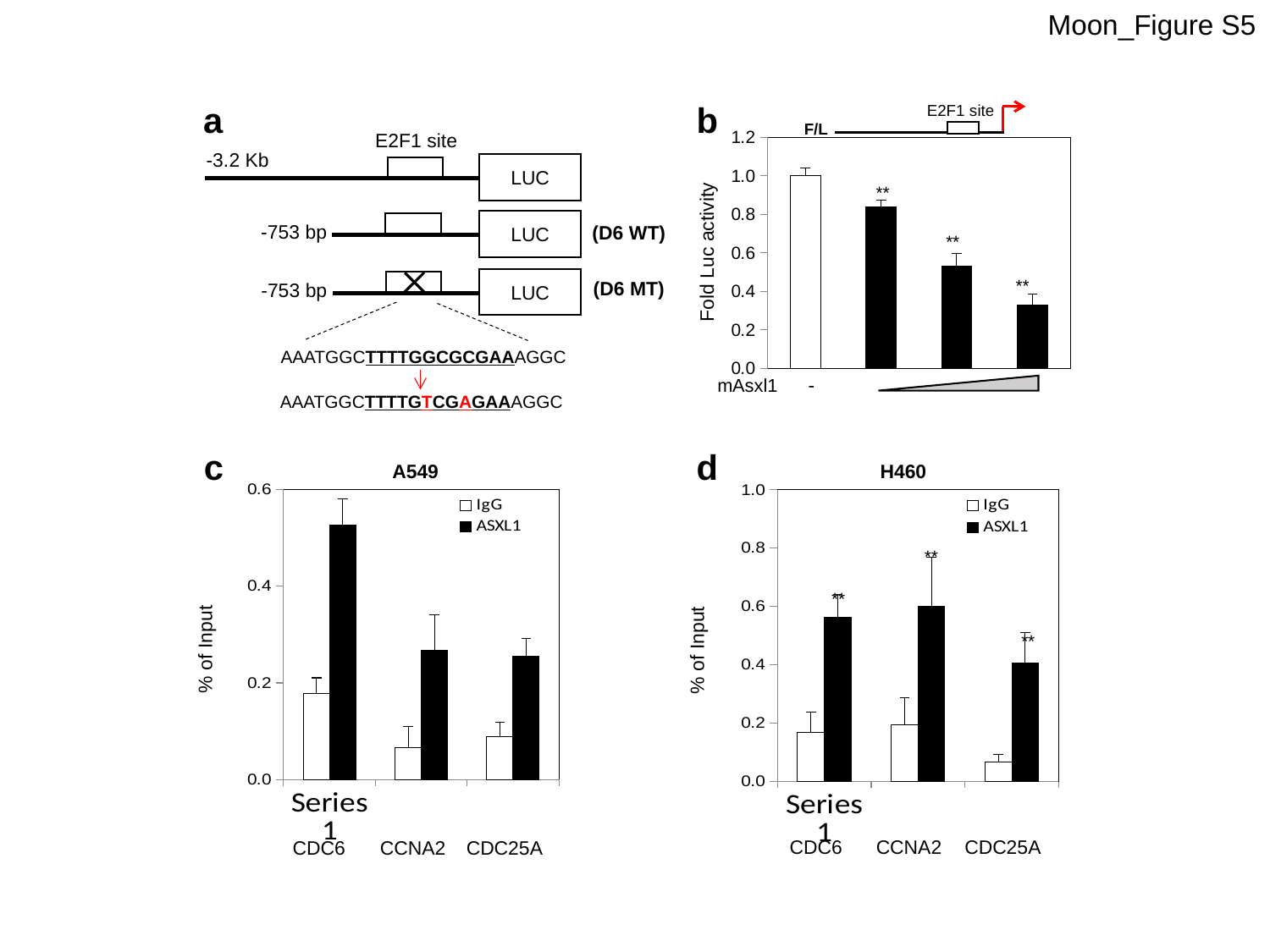

Moon_Figure S5
a
b
E2F1 site
F/L
E2F1 site
### Chart
| Category | 열1 |
|---|---|
| | 1.0 |
| | 0.8409835 |
| | 0.53 |
| | 0.328 |-3.2 Kb
LUC
**
LUC
-753 bp
(D6 WT)
**
Fold Luc activity
**
LUC
(D6 MT)
-753 bp
AAATGGCTTTTGGCGCGAAAGGC
mAsxl1 -
AAATGGCTTTTGTCGAGAAAGGC
c
d
A549
H460
### Chart
| Category | IgG | ASXL1 |
|---|---|---|
| | 0.17872181925171127 | 0.5260879315426742 |
| | 0.06670978759273977 | 0.2669502787677927 |
| | 0.08825504225403001 | 0.25443061982937676 |
### Chart
| Category | IgG | ASXL1 |
|---|---|---|
| | 0.16678068899331544 | 0.5618942263331504 |
| | 0.19342345179057593 | 0.5996471460363326 |
| | 0.06779754987630059 | 0.4062609579860991 |**
***
**
% of Input
% of Input
**
**
**
CDC6
CCNA2
CDC25A
CDC6
CCNA2
CDC25A

## Slide 11
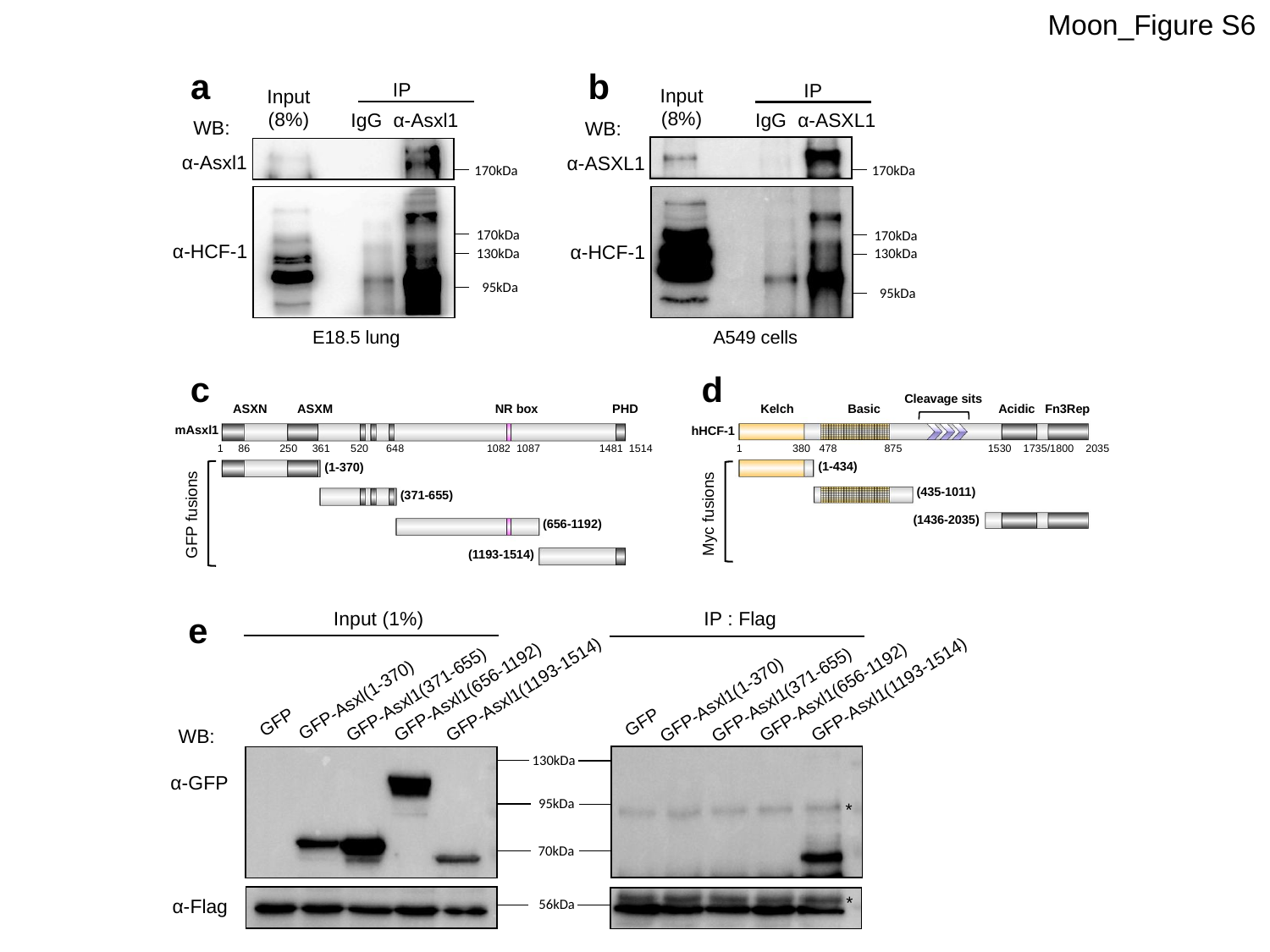

Moon_Figure S6
a
b
IP
IgG α-Asxl1
IP
IgG α-ASXL1
Input
(8%)
WB:
α-ASXL1
170kDa
170kDa
α-HCF-1
130kDa
95kDa
A549 cells
Input
(8%)
WB:
α-Asxl1
170kDa
170kDa
α-HCF-1
130kDa
95kDa
E18.5 lung
c
d
Cleavage sits
ASXN ASXM NR box PHD
Kelch Basic Acidic Fn3Rep
mAsxl1
hHCF-1
1 86 250 361 520 648 1082 1087 1481 1514
1 380 478 875 1530 1735/1800 2035
(1-434)
(1-370)
(435-1011)
(371-655)
Myc fusions
GFP fusions
(1436-2035)
(656-1192)
(1193-1514)
Input (1%)
IP : Flag
GFP-Asxl1(1193-1514)
GFP-Asxl1(656-1192)
GFP-Asxl1(371-655)
GFP-Asxl1(1-370)
GFP
e
GFP-Asxl1(1193-1514)
GFP-Asxl1(656-1192)
GFP-Asxl1(371-655)
GFP-Asxl(1-370)
GFP
WB:
α-GFP
130kDa
95kDa
*
70kDa
*
α-Flag
56kDa

## Slide 12
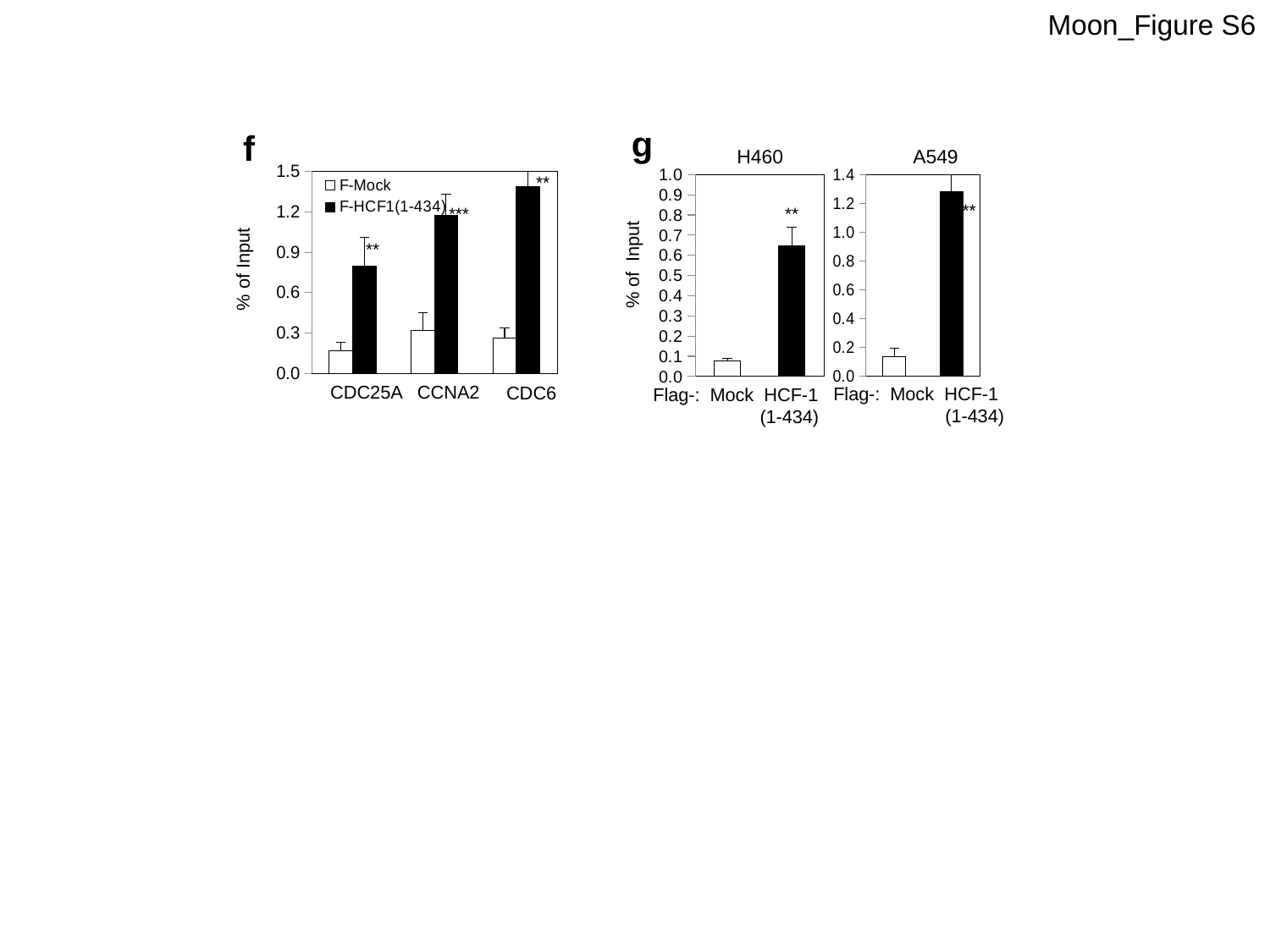

Moon_Figure S6
g
f
H460
A549
### Chart
| Category | F-Mock | F-HCF1(1-434) |
|---|---|---|
| | 0.1687119215545526 | 0.7983757894453741 |
| | 0.31765880449157896 | 1.170384564092976 |
| | 0.2631331463779891 | 1.3865840757237682 |
### Chart
| Category | 열1 |
|---|---|
| Flag | 0.07671639430197445 |
| Flag-HCF1(1-434) | 0.6464116511202859 |
### Chart
| Category | 열1 |
|---|---|
| IgG | 0.13440856036255786 |
| Asxl1 | 1.2807131078938128 |**
**
**
***
% of Input
% of Input
**
CCNA2
CDC25A
CDC6
Flag-: Mock HCF-1
 (1-434)
Flag-: Mock HCF-1
 (1-434)

## Slide 13
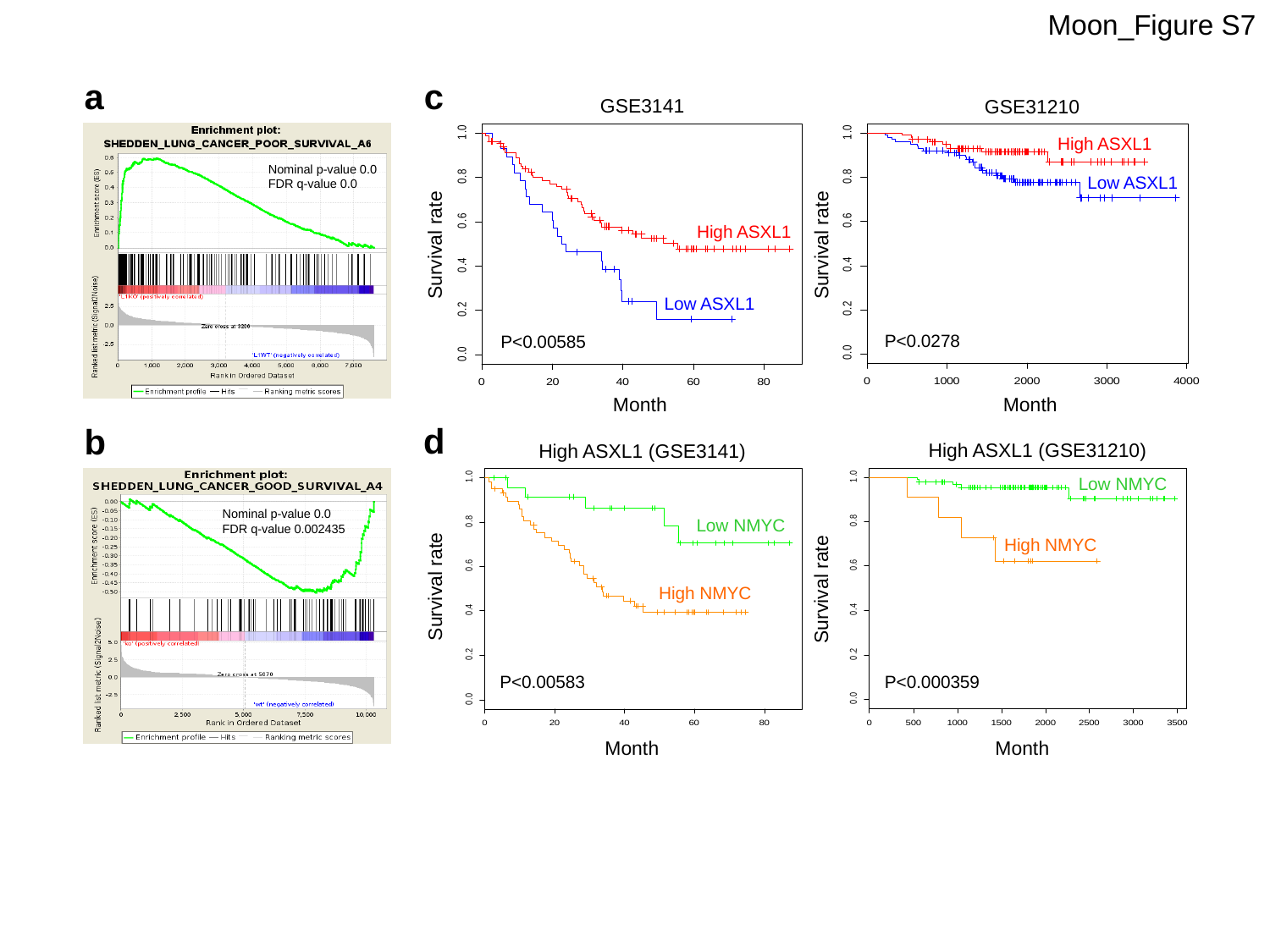

Moon_Figure S7
a
c
GSE3141
GSE31210
Nominal p-value 0.0
FDR q-value 0.0
High ASXL1
Low ASXL1
High ASXL1
Survival rate
Survival rate
Low ASXL1
P<0.0278
P<0.00585
Month
Month
d
b
High ASXL1 (GSE31210)
High ASXL1 (GSE3141)
Low NMYC
Nominal p-value 0.0
FDR q-value 0.002435
Low NMYC
High NMYC
Survival rate
Survival rate
High NMYC
P<0.000359
P<0.00583
Month
Month
