# Supplementary material for: Asxl1 exerts an antiproliferative effect on mouse lung maturation via epigenetic repression of the E2f1-Nmyc axis
Source: Cell Death Dis. 2018 Nov 2;9(11):1118. doi: 10.1038/s41419-018-1171-z (PMC6215009; doi:10.1038/s41419-018-1171-z)
Supplement: Supplementary file 3 — Supplementary Table S1 [file 41419_2018_1171_MOESM3_ESM.docx]

**Supplementary Table S1.** Analysis of progeny from intercrosses of *Asxl1^+/-^* mice

|  | | Genotypes | | |
| --- | --- | --- | --- | --- |
| Embryonic stages | Embryos | *Asxl1^+/+^* | *Asxl1^+/-^* | *Asxl1^-/-^* |
| E14.5 | 132 | 35 | 74 | 23 |
| E16.5 | 27 | 7 | 14 | 6 |
| E18.5 | 205 | 66 | 94 | 45 |
| P0 | 66 | 18 | 31 | 17 |
| P1 | 49 | 18 | 31 | 0 |
| Total | 479 | 144 | 244 | 91 |
