# Supplementary material for: Asxl1 exerts an antiproliferative effect on mouse lung maturation via epigenetic repression of the E2f1-Nmyc axis
Source: Cell Death Dis. 2018 Nov 2;9(11):1118. doi: 10.1038/s41419-018-1171-z (PMC6215009; doi:10.1038/s41419-018-1171-z)
Supplement: Supplementary file 5 — Supplementary Table S3 [file 41419_2018_1171_MOESM5_ESM.pdf]

Supplementary Table S3. Comparison of the gene expression patterns from Asxl1-/-, Nmyc KO and Nmyc TG data (Change of log).

Asxl1-/-/ WT Microarray			Nmyc9a(KO)/ WT_JR Microarray			SftpC-Nmyc1(TG)/ WT_BH Microarray		
name	Gene Symbol	$Asxl1^{-/-}$/ WT Microarray	Gene Symbol	Regulation	Nmyc9a/ WT_JR Microarray	Gene Symbol	Regulation	SftpC-Nmyc1/ WT_BH Microarray
ubiquitin-like, containing PHD and RING finger domains, 1	Uhrf1	1.3	Uhrf1	positive	-1.2	Uhrf1	positive	2.3
CDC28 protein kinase regulatory subunit 2	Cks2	1.4	Cks2	positive	-1.2	Cks2	positive	1.4
	Rrm2	1.2	Rrm2	positive	-1.0	Rrm2	positive	1.6
deoxythymidylate kinase	Dtymk	1.2	Dtymk	positive	-1.2	Dtymk	positive	1.2
ribonucleotide reductase M1	Rrm1	1.0	Rrm1	positive	-0.8	Rrm1	positive	1.2
proliferating cell nuclear antigen	Pcna	0.9	Pcna	positive	-1.1	Pcna	positive	1.4
HLA-B-associated transcript 1A	Bat1a	0.4	Bat1a	positive	-0.1	Bat1a	positive	0.5
RNA binding motif protein, X chromosome retrogene	Rbmxrt	0.7	Rbmxrt	positive	-0.7	Rbmxrt	positive	1.2
flap structure specific endonuclease 1	Fen1	0.8	Fen1	positive	-0.9	Fen1	positive	1.5
THO complex 4	Thoc4	0.8	Thoc4	positive	-0.6	Thoc4	positive	1.3
	Dtymk	0.8	Dtymk	positive	-1.2	Dtymk	positive	1.2
exportin 1, CRM1 homolog (yeast)	Xpo1	0.7	Xpo1	positive	-0.7	Xpo1	positive	0.7
nucleolin	Ncl	0.4	Ncl	positive	-0.4	Ncl	positive	1.5
FK506 binding protein 3	Fkbp3	0.7	Fkbp3	positive	-0.7	Fkbp3	positive	1.4
splicing factor proline	Sfpq	0.6	Sfpq	positive	-0.3	Sfpq	positive	0.9
chromatin licensing and DNA replication factor 1	Cdt1	0.7	Cdt1	positive	-0.7	Cdt1	positive	2.0
profilin 2	Pfn2	0.7	Pfn2	positive	-1.1	Pfn2	positive	0.7
DnaJ (Hsp40) homolog, subfamily C, member 9	Dnajc9	0.6	Dnajc9	positive	-1.1	Dnajc9	positive	1.4
high mobility group box 1	Hmgb1	0.4	Hmgb1	positive	-0.5	Hmgb1	positive	0.5
nucleophosmin 1	Npm1	0.5	Npm1	positive	-0.5	Npm1	positive	1.8
Sjogren syndrome antigen B	Ssb	0.5	Ssb	positive	-0.3	Ssb	positive	0.8
MYB binding protein (P160) 1a	Mybbp1a	0.5	Mybbp1a	positive	-0.2	Mybbp1a	positive	1.6

proteasome (prosome, macropain) subunit, alpha type 1	Psma1	0.5	Psma1	positive	-0.5	Psma1	positive	0.7
smu-1 suppressor of mec-8 and unc-52 homolog (C. elegans)	Smu1	0.3	Smu1	positive	-0.4	Smu1	positive	0.4
NADH dehydrogenase (ubiquinone) 1 alpha subcomplex, 4	Ndufa4	0.4	Ndufa4	positive	-0.7	Ndufa4	positive	0.7
SET and MYND domain containing 2	Smyd2	0.4	Smyd2	positive	-0.7	Smyd2	positive	1.6
pleiotropic regulator 1, PRL1 homolog (Arabidopsis)	Plrg1	0.3	Plrg1	positive	-0.5	Plrg1	positive	0.7
structure specific recognition protein 1	Ssrp1	0.3	Ssrp1	positive	-0.4	Ssrp1	positive	1.0
ribosomal protein L13a	Rpl13a	0.3	Rpl13a	positive	-0.3	Rpl13a	positive	0.8
THUMP domain containing 1	Thumpd1	0.3	Thumpd1	positive	-0.4	Thumpd1	positive	1.0
polypyrimidine tract binding protein 1	Ptbp1	0.1	Ptbp1	positive	-0.3	Ptbp1	positive	0.6
tyrosine 3-monooxygenase	Ywhaq	0.2	Ywhaq	positive	-0.6	Ywhaq	positive	0.5
spectrin beta 2	Spnb2	-0.4	Spnb2	negative	0.7	Spnb2	negative	-0.9
nuclear receptor coactivator 5	Ncoa5	0.2	Ncoa5	positive	-0.2	Ncoa5	positive	0.5
coiled-coil-helix-coiled-coil-helix domain containing 2	Chchd2	0.1	Chchd2	positive	-0.4	Chchd2	positive	0.3
signal transducer and activator of transcription 3	Stat3	-0.8	Stat3	negative	0.3	Stat3	negative	-0.5
