# Supplementary material for: Asxl1 exerts an antiproliferative effect on mouse lung maturation via epigenetic repression of the E2f1-Nmyc axis
Source: Cell Death Dis. 2018 Nov 2;9(11):1118. doi: 10.1038/s41419-018-1171-z (PMC6215009; doi:10.1038/s41419-018-1171-z)
Supplement: Supplementary file 6 — Supplementary Table S4 [file 41419_2018_1171_MOESM6_ESM.pdf]

Supplementary Table S4. List of ASXL1-associated proteins*

Protein	Description	Mass (kDa)	Peptide count	Coverage (%)
HCF1	Host cell factor 1	209	17	16
ASXL1	Additional sex comb-like 1	165	10	7
OGT	UDP-N-acetylglucosamine peptide N-acetylglucosaminyltransferase	117	6	9
SCYL2	SCY1-like protein 2: CVAK104	104	10	16
TIF1b	Transcription intermediary factor 1-beta: TRIM28	89	7	13
BAP1	BRCA1-associated protein 1	80	8	15
FOXK1	Forkhead box protein K1	75	7	19
GRP78	78 kDa glucose-regulated protein: BIP	72	10	22
PRMT5	Protein arginine N-methyltransferase 5 isoform b	71	8	19
HSP70	Heat shock 70 kDa protein	70	15	31
PKM2	Pyruvate kinase isozyme M2	58	9	24
TCP1b	T-complex protein 1 subunit beta	57	6	18
STK38	Serine/threonine-protein kinase 38: nuclear Dbf2-related kinase 1	54	6	22
PPM1B	Protein phosphatase 1B: PPM1B, PP2Cbeta	53	6	17
Reptin	RuvB-like 2: RVB2	51	7	22
TUBB	Tubulin beta	50	17	50
TUBA	Tubulin alpha	50	13	39
Potin	RuvB-like 1: RVB1	50	6	20
HNRPH	Heterogeneous nuclear ribonucleoprotein H	49	5	19
ENO1L1	Alpha-enolase	47	6	27
Actin	Actin, cytoplasmic 1	42	9	34
WD45	Methylosome protein 50: WD45/MEP50	37	2	11
RACK1	Guanine nucleotide-binding protein subunit beta-2-like 1: GNB2L1	35	4	16
ANT2	ADP/ATP translocase 2: SLC25A5	33	3	12
RAN	GTP-binding nuclear protein Ran	24	3	15
RAB5C	Ras-related protein Rab-5C	23	3	16
PRDX1	Peroxiredoxin-1	22	3	16
CBX5	Chromobox protein homolog 3: CBX5, HP1a	21	3	23
H2A	Histone H2A	14	3	27
H4	Histone H4	11	2	21

*>peptide count 5 or >coverage 10%, - keratin, - ribosomal protein
